# *TRPV6*-related pancreatitis: natural history and the impact of the pancreas-specific deletion on pancreatitis in mice

**DOI:** 10.1007/s00535-025-02323-y

**Published:** 2025-11-17

**Authors:** Atsushi Masamune, Emmanuelle Masson, Wen-Bin Zou, Agnieszka Magdalena Rygiel, Sudipta Dhar Chowdhury, Kazuhiro Kikuta, Hidehiro Hayashi, Akira Sasaki, Hitomi Nakasuji, Ryotaro Matsumoto, Tetsuya Takikawa, Yan Xu, Ren Jie, Yasumasa Sekino, Toshiaki Abe, Waku Hatta, Tetsuya Niihori, Yoko Aoki, Reiko Sakaguchi, Yasuo Mori, Vinciane Rebours, Louis Buscail, Yuan-Chen Wang, Reuben Thomas Kurien, Sandhya S. Visweswariah, Jonas Rosendahl, Claude Ferec, Grzegorz Oracz, Heiko Witt, Zhuan Liao, Jian-Min Chen, Shin Hamada

**Affiliations:** 1 Division of Gastroenterology, Tohoku University Graduate School of Medicine, Sendai, Japan; 2 Univ Brest, Inserm, EFS, UMR 1078, GGB, Brest, France; 3 Service de Génétique Médicale et de Biologie de la Reproduction, CHU Brest, Brest, France; 4 Department of Gastroenterology, National Clinical Research Center for Digestive Diseases, Changhai Hospital, Naval Medical University, Shanghai, 200433 China; 5 Department of Medical Genetics, Laboratory of Hereditary Diseases, Institute of Mother and Child, Warsaw, Poland; 6 Department of Gastroenterology, Christian Medical College Vellore, Vellore, Tamil Nadu India; 7 Department of Medical Genetics, Tohoku University Graduate School of Medicine, Sendai, Japan; 8 Laboratory of Biomaterials and Chemistry, School of Medicine, University of Occupational and Environmental Health, Kitakyushu, Japan; 9 Laboratory of Molecular Biology, Department of Synthetic Chemistry and Biological Chemistry, Graduate School of Engineering, Kyoto University, Kyoto, Japan; 10 Pancreatology and Digestive Oncology Department, Beaujon Hospital, APHP Clichy, Université Paris Cité, 92110 Paris, France; 11 Department of Gastroenterology and Pancreatology, CHU Rangueil and University of Toulouse, Toulouse, France; 12 Department of Developmental Biology and Genetics, Indian Institute of Science, Bangalore, India; 13 Department of Internal Medicine I, Martin Luther University, Halle (Saale), Germany; 14 Department of Gastroenterology, Hepatology, Feedings Disorders and Pediatrics, The Children’s Memorial Health Institute, Warsaw, Poland; 15 Pediatric Nutritional Medicine & Else Kröner-Fresenius-Centre for Nutritional Medicine EKFZ, Technical University Munich TUM, Freising, Germany

**Keywords:** Acute pancreatitis, Calcium channel, Chronic pancreatitis, Pancreatic cancer, Pancreatic exocrine insufficiency, Transient receptor potential

## Abstract

**Background:**

The transient receptor potential cation channel subfamily V member 6 (*TRPV6*) gene, encoding a calcium-selective ion channel, was recently identified as a susceptibility gene for pancreatitis. This study aimed to clarify the natural history of *TRPV6*-related pancreatitis and the impact of pancreas-specific deletion of *Trpv6* on pancreatitis in mice.

**Methods:**

Clinical information of the patients carrying functionally impaired *TRPV6* variants, defined by Ca^2+^ imaging and minigene assays, was collected from six international centers. Cumulative rates were assessed using Kaplan–Meier analysis. As controls, Japanese patients with alcohol-unrelated pancreatitis carrying pathogenic variants in *PRSS1* or *SPINK1*, as well as those without pathogenic variants in pancreatitis susceptibility genes, were enrolled. A pancreas-specific *Trpv6* conditional knockout mouse was established by crossing the *Trpv6* floxed mouse and the *Pdx-1-Cre* mouse. Pancreatitis was induced by repeated intraperitoneal injections of caerulein.

**Results:**

Ninety-four patients with functionally impaired *TRPV6* variants, including six splice-site variants, were enrolled. The median age at symptom onset was 16 years. The cumulative rates of pancreatic calcification, pancreatic exocrine insufficiency, diabetes mellitus, and interventions for pancreatitis were 55.5%, 20.1%, 10.8%, and 41.6% at 30 years, and 81.5%, 49.6%, 45.4%, and 69.9% at 50 years, respectively. Pancreas-specific *Trpv6* knockout mice developed more severe acute and chronic pancreatitis than the control mice. Caerulein treatment increased the TRPV6 expression in pancreatic acinar cells.

**Conclusions:**

Functionally impaired *TRPV6* variants significantly influenced the clinical outcomes of chronic pancreatitis. TRPV6 in pancreatic acinar cells might play a protective role against pancreatitis in mice.

**Supplementary Information:**

The online version contains supplementary material available at 10.1007/s00535-025-02323-y.

## Introduction

Chronic pancreatitis (CP) is a pathological fibro-inflammatory syndrome of the pancreas, characterized by irreversible morphological changes, pain, and the eventual loss of exocrine and endocrine functions of the organ [1–4]. CP develops resulting from the interactions between genetic factors and environmental ones, such as alcohol and smoking. Since the landmark identification of mutations in the *PRSS1*, which encodes cationic trypsinogen, as a cause of hereditary pancreatitis in 1996 [5], several susceptibility genes for pancreatitis have been identified, including *CFTR*, which encodes cystic fibrosis transmembrane conductance regulator [6, 7], *SPINK1*, which encodes serine protease inhibitor Kazal type 1 [8], *CTRC*, which encodes chymotrypsin C [9], and *CPA1*, which encodes carboxypeptidase A1 [10]. Main mechanistic pathways that explain the pathogenic effects of variants in these susceptibility genes include trypsin-dependent (represented by *PRSS1*, *SPINK1*, and *CTRC*), misfolding-dependent (represented by *CPA1*), and ductal pathways (represented by *CFTR*) [2].

In 2020, we reported that functionally impaired variants in the transient receptor potential cation channel subfamily V member 6 (*TRPV6*) gene, which encodes a calcium channel, are overrepresented in patients with alcohol-unrelated CP in Japan, France, and Germany [11]. Subsequent studies confirmed the association with functionally impaired *TRPV6* variants in Chinese patients with CP [12], French patients with hereditary and familial pancreatitis [13], and Polish patients with alcohol-unrelated early-onset CP [14]. A novel frameshift *TRPV6* variant was identified in an Indian family with hereditary pancreatitis [15]. However, little is known about the natural history of patients with *TRPV6*-related pancreatitis, partly because the number of such patients in one population is limited.

TRPV6 is a highly Ca^2+^-selective ion channel that regulates apical Ca^2+^ entry in absorptive and secretory tissues and plays a central role in Ca^2+^ homeostasis in the body [16]. TRPV6 is expressed in various mouse tissues, including the intestine, where it plays a role in Ca^2+^ absorption [17]. *Trpv6* knockout mice exhibit physiological problems such as defective intestinal Ca^2+^ absorption, male infertility, and excessive urine Ca^2+^ excretion [18, 19]. We have previously reported that *Trpv6*^mut/mut^ mice (homozygous for p.Asp581Ala) developed more severe caerulein-induced pancreatitis than control mice [11]. However, mice with a global *Trpv6* knockout may exhibit altered systemic Ca^2+^ homeostasis, which could contribute to the exacerbation of pancreatitis. Here, we conducted an international multicenter study to clarify the clinical outcomes of *TRPV6*-related pancreatitis. In addition, we developed pancreas-specific *Trpv6* conditional knockout (CKO) mice to clarify the role of pancreatic *Trpv6* in pancreatitis.

## Methods

### Participants

This retrospective international multicenter study involved centers in Japan, China, France, Germany, Poland, and India. It was conducted in accordance with the guidelines of the Declaration of Helsinki and approved by the Institutional Review Board of the participating institutions. All study participants and/or their legal guardians provided written informed consent prior to study enrollment.

### Genetic analysis

Genetic analyses for all exons and flanking introns of *TRPV6*, *PRSS1*, *SPINK1*, *CFTR*, *CTRC*, and *CPA1* were performed by direct sequencing, target sequencing, or whole-exome sequencing as previously described [8–11, 20]. The NM_018646.5 GenBank reference sequence was used for *TRPV6*.

### Mutagenesis

*TRPV6* mutant constructs were prepared using the KOD-Plus-Mutagenesis Kit (TOYOBO, Osaka, Japan) according to the manufacturer’s instructions. Successful mutagenesis was confirmed by direct sequencing. The construction of the full-length wild-type TRPV6 and the ancestral haplotype expression vectors in pcDNA3.1(−) was reported previously [21].

### Ca^2+^ imaging assay

To evaluate the impact of the identified nonsynonymous variants on TRPV6 channel activity, we performed a Ca^2+^ imaging assay as previously described [11]. Briefly, HEK293 cells (American Type Culture Collection, Manassas, VA) were co-transfected with recombinant TRPV6 expression plasmids and either pEGFP-N1 or pEGFP-C1 (Clontech Laboratories, Palo Alto, CA) as a transfection marker, using SuperFect transfection reagent (Qiagen, Hilden, Germany). Following transfection, cells were loaded with 1 μmol/L fura-2-acetoxymethyl ester (Fura-2-AM; Dojindo, Kumamoto, Japan). Fluorescence images were acquired and analyzed using a video image analysis system (AQUACOSMOS; Hamamatsu Photonics, Hamamatsu, Japan). Fura-2-AM fluorescence at an emission wavelength of 510 nm was recorded upon sequential excitation at 340 and 380 nm. The increase in the fluorescence ratio (F340/F380) induced by 2 mmol/L Ca^2+^ was assessed. TRPV6 activity in cells expressing wild-type TRPV6 was defined as 100%. Variants were considered functionally impaired when the increase in [Ca^2+^]_i_ was significantly reduced compared with the wild type.

### Minigene assay

The BAC clone RP11-520H11, containing the full-length human *TRPV6* gene, was purchased from Advanced GenoTechs Co. (Tsukuba, Japan). Gene fragments spanning the flanking exons were amplified using the primer sets listed in Supplementary Table 1, KOD FX DNA polymerase (TOYOBO, Osaka, Japan), and RP11-520H11 as the template. Except for the construction of the minigene containing the c.2015+2T>C variant, a stop codon was introduced at the 5′ end of the reverse primers. The PCR products were subcloned with the Zero Blunt TOPO PCR Cloning Kit (Thermo Fisher Scientific, Waltham, MA), and patient-derived variants were introduced using the KOD-Plus-Mutagenesis Kit (TOYOBO). Following digestion with *EcoRI* and *XhoI* (Nippon Gene, Tokyo, Japan), the amplified fragments were subcloned into the multiple cloning site of the pcDNA3.1/V5-His A vector (Thermo Fisher Scientific). All constructs were verified by direct sequencing.

### In vitro splicing assay

HEK293T cells (American Type Culture Collection) were transfected with 2 μg of either wild-type or variant minigene-containing pcDNA3.1/V5-His A vectors using Lipofectamine 2000 (Thermo Fisher Scientific). After 24 h, total RNA was extracted using the RNeasy Kit (Qiagen). One microgram of RNA was reverse-transcribed with the SuperScript VILO Master Mix (Thermo Fisher Scientific), followed by PCR amplification using the respective primer sets (Supplementary Table 2). PCR products were separated by agarose gel electrophoresis, purified with the QIAquick Gel Extraction Kit (Qiagen), and sequenced with T7 or SP6 primers after TA cloning (Zero Blunt TOPO PCR Cloning Kit; Thermo Fisher Scientific).

### Data collection

We collected clinical information from participating institutions using a standardized case report form. CP was diagnosed according to the diagnostic criteria used in the respective countries, based on symptoms such as abdominal pain, pancreatic exocrine insufficiency (PEI), history of acute pancreatitis, and imaging findings of the pancreas on computed tomography and/or magnetic resonance imaging [22, 23] (Supplementary Table 3). Patients with recurrent acute pancreatitis (RAP) who did not meet the diagnostic criteria for CP were classified as RAP. Patients with alcohol-related pancreatitis were excluded. PEI was diagnosed based on clinical steatorrhea, the need for long-term oral pancreatic enzyme supplementation, and/or abnormal pancreatic exocrine function tests (fecal elastase-1 or *N*-benzoyl-l-tyrosyl-*p*-aminobenzoic acid test) [22, 24, 25] (Supplementary Table 3). DM was diagnosed when fasting plasma glucose exceeded 126 mg/dL, casual plasma glucose exceeded 200 mg/dL, HbA1c was ≥ 6.5%, or 2-h plasma glucose exceeded 200 mg/dL during a 75-g oral glucose tolerance test [26].

To compare the natural history across variant statuses—*PRSS1*-related pancreatitis, *SPINK1*-related pancreatitis, and PV-negative pancreatitis (defined as the absence of pathogenic variants (PVs) in *TRPV6*, *PRSS1*, *SPINK1*, *CTRC*, and *CPA1*)—we enrolled Japanese patients with alcohol-unrelated RAP or CP who underwent genetic testing at Tohoku University Hospital.

### Establishment of the pancreas-specific *Trpv6* knockout mouse

All animal experiments were approved by the Institution’s Animal Care and Use Committee and conducted in accordance with the regulations for animal experiments and related activities at Tohoku University (article No. 2018MdLMO-177-08). *Pdx-1-Cre* transgenic mice were obtained from the National Cancer Institute Mouse Repository (Frederick, MD). *Trpv6* floxed mouse was established by introducing *loxP* sites flanking exons 13–15 of *Trpv6* using genome editing. These mice were crossed with the *Pdx-1-Cre* mouse to obtain the *Pdx-1-Cre*::*Trpv6*^floxed/floxed^ (*Trpv6* CKO) mouse, in which exons 13–15 of *Trpv6* are specifically deleted in the pancreas.

Genomic DNA was prepared from each mouse organ using the PureLink Genomic DNA Mini Kit (Thermo Fisher Scientific). We performed PCR to confirm *Cre*-dependent recombination at the *Trpv6* locus, with primer sequences and PCR conditions listed in Supplementary Table 4.

### Induction of pancreatitis

*Trpv6* CKO mice and the control *Trpv6* floxed mice aged 3 months received 8-hourly intraperitoneal injections of normal saline or the cholecystokinin analog caerulein (100 μg/kg body weight; Selleck Biotechnology, Yokohama, Japan) for two consecutive days [27]. Serum amylase level was measured using the Fuji Dri-Chem 7000 analyzer (FUJIFILM Corporation, Tokyo, Japan). Pancreatic tissues were removed, fixed in 4% paraformaldehyde (FUJIFILM Wako Pure Chemical), embedded in paraffin wax for hematoxylin and eosin (H&E) staining and Sirius Red staining [28]. Histological findings were evaluated (Supplementary Table 5), and immunohistochemical staining for TRPV6 was performed as previously described [29, 30]. Slides were boiled in target retrieval solution (Dako, Glostrup, Denmark) and incubated with rabbit anti-TRPV6 antibody (ACC-036; Alomone Labs, Jerusalem, Israel) overnight at 4 °C. Immunoreactivity was visualized using a streptavidin–biotin–peroxidase complex detection kit (Histofine Kit; Nichirei Biosciences Inc., Tokyo, Japan) and diaminobenzidine (Dojindo).

CP was induced by 6-hourly intraperitoneal injections of caerulein (100 μg/kg body weight), 3 days/week for four consecutive weeks [31].

### Establishment of pancreatic organoids

Organoids were established from the pancreas of *Trpv6* CKO mice and the *Trpv6* floxed mice as previously described with minor modifications [32, 33]. Pancreatic tissue was minced, digested with Collagenase P (Roche, Mannheim, Germany), and embedded in Matrigel Matrix (Corning, Corning, NY). The cultures were overlaid with advanced DMEM/F-12 medium supplemented with the reagents listed in Supplementary Table 6.

Organoids were exposed to various concentrations of Ca^2+^ and evaluated morphologically. CFTR activity was assessed using the forskolin-induced swelling assay, which reflects CFTR-mediated anion secretion and fluid influx into the organoid [34]. Organoids were either left untreated or treated with 10 μmol/L forskolin for 16 h. Their diameters were measured using a BZ-9000 microscope (Keyence, Osaka, Japan) and analyzed with the BZ-II analyzer (Keyence). The relative organoid size was defined as the cross-sectional area after forskolin treatment divided by that before treatment.

### Quantitative real-time PCR

Eight-week-old male C57BL/6J mice (Jackson Laboratory Japan, Inc., Yokohama, Japan) received eight hourly intraperitoneal caerulein injections (100 μg/kg body weight) or saline for one or two consecutive days. Total RNA was extracted using the RNeasy kit, and reverse-transcribed using the SuperScript VILO Master Mix. Gene expression was quantified by real-time PCR using the StepOnePlus system (Thermo Fisher Scientific) with Fast SYBR Green Master Mix (Thermo Fisher Scientific) and the primer sets listed in Supplementary Table 7.

### Statistical analysis

For Ca^2+^ imaging assays, data are presented as mean ± standard error of the mean from 2 or 3 independent transfections, and the differences among groups were analyzed using the Tukey–Kramer method. For clinical data, missing values were excluded from the analysis. Kaplan–Meier survival analysis was used to estimate the cumulative incidence rates of symptom onset, complications, and treatments, with 95% confidence intervals (CIs). Cumulative rates over time were compared with the log-rank test. The differences in histological findings were analyzed using the unpaired *t*-test. A two-sided *P* value of < 0.05 was considered statistically significant. All statistical analyses were performed with SPSS Statistics (version 20.0; IBM Corp., Armonk, NY) and R (version 4.2.1, R Foundation for Statistical Computing, Vienna, Austria).

## Results

### Identification of novel nonsynonymous and splice-site *TRPV6* variants

In addition to the 41 *TRPV6* variants previously reported to be functionally impaired based on Ca^2+^ imaging assays [11–15], we identified four nonsynonymous *TRPV6* variants of unknown functional consequence (Table 1). The c.715_724del [p.Val239SerfsTer53] variant was detected in two German, one French, and one Polish patients with pancreatitis; the c.1137C>A [p.Tyr379Ter] variant was identified in a Chinese patient; the c.1759_1761del [p.Tyr587del] variant in a Japanese patient; and the c.1870C>T [p.Arg624Ter] variant in two Austrian, one French, and one Japanese patient. In addition, six splice-site variants were identified: c.347-2A>G, c.469+1G>C, c.1029+1G>A, and c.1407-2A>G variants in French patients (one each), c.607+5G>C in a Japanese patient, and c.2015+2T>C in a Chinese patient.

**Table 1:** Non-synonymous *TRPV6* variants detected in patients in this study

Exon	Nucleotide change	Amino acid change	*n*	Nationality	Reference for functional assay
1	c.75_81delGGTCTGG	p.Arg25SerfsTer25	3	3FR	Hamada [13]
1	c.245delA	p.Lys82ArgfsTer10	3	3FR	Masamune [11]
2	c.303delG	p.Asn102ThrfsTer24	1	1FR	Masamune [11]
4	c.515T>C	p.Leu172Pro	1	1CH	Zou [12]
4	c.520C>T	p.Arg174Ter	2*	1JP, 1PO	Masamune [11]
5	c.629C>T	p.Ala210Val	2	2JP	Masamune [11]
6	c.715_724delGTGTTACAC	p.V239SfsTer53	4	2GM, 1FR, 1PO	Not previously reported
6	c.786C>G	p.Tyr262Ter	1	1JP	Masamune [11]
6	c.802C>A	p.His268Asn	1	1CH	Zou [12]
7	c.932G>T	p.Gly311Val	2	2FR	Masamune [11]
7	c.1025G>A	p.Arg342Gln	5	3PO, 1FR, 1GM	Masamune [11]
8	c.1033C>T	p.Arg345Cys	3	1FR, 1JP, 1PO	Masamune [11]
8	c.1034G>A	p.Arg345His	8	5CH, 2FR, 1GM	Masamune [11]
8	c.1094G>A	p.Gly365Glu	1	1GM	Masamune [11]
8	c.1137C>A	p.Tyr379Ter	1	1CH	Not previously reported
8	c.1155G>A	p.Met385Ile	1	1CH	Zou [12]
8	c.1168C>T	p.Arg390Cys	2	2FR	Hamada [13]
8	c.1174C>T	p.Leu392Phe	2	2JP	Masamune [11]
8	c.1207C>T	p.Arg403Trp	1	1FR	Hamada [13]
9	c.1273C>T	p.Arg425Trp	1	1CH	Zou [12]
9	c.1274G>A	p.Arg425Gln	2	1FR, 1JP	Masamune [11]
9	c.1282G>A	p.Gly428Arg	2	2JP	Masamune [11]
11	c.1417G>A	p.Ala473Thr	1	1CH	Zou [12]
11	c.1447C>T	p.Arg483Trp	2	2GM	Masamune [11]
11	c.1448G>A	p.Arg483Gln	4	2JP, 1CH, 1GM	Masamune [11]
11	c.1465G>A	p.Gly489Arg	1	1CH	Masamune [11]
11	c.1474_1475delGT	p.Val492ThrfsTer136	3	3IN	Shah [15]
11	c.1517T>C	p.Met506Thr	2	1CH, 1FR	Zou [12]
11	c.1521C>A	p.Tyr507Ter	1	1CH	Zou [12]
12	c.1612G>T	p.Ala538Thr	1	1FR	Hamada [13]
12	c.1618G>T	p.V540Phe	1	1JP	Masamune [11]
13	c.1723G>A	p.E575Lys	6	5FR, 1CH	Masamune [11]
13	c.1727T>G	p.Leu576Arg	1	1PO	Oracz [14]
13	c.1738A>T	p.Ile580Phe	1	1JP	Masamune [11]
13	c.1759_1761delTAC	p.Tyr587del	1	1JP	Not previously reported
13	c.1816G>A	p.Ala606Thr	1	1JP	Masamune [11]
13	c.1823T>G	p.Leu608Arg	1	1JP	Masamune [11]
13	c.1825C>T	p.Leu609Phe	1	1JP	Masamune [11]
13	c.1841T>C	p.Leu614Pro	1	1FR	Hamada [13]
13	c.1844T>C	p.Ile615Thr	1	1FR	Hamada [13]
13	c.1864_1867delCACT	p.His622GlyfsX20	1	1GM	Masamune [11]
13	c.1870C>T	p.Arg624Ter	4	2AU, 1FR, 1JP	Not previously reported
13	c.1876G>C	p.Ala626Pro	1	1GM	Masamune [11]
14	c.1936C>T	p.Arg646Trp	1*	1FR	Hamada [13]
14	c.1977C>A	p.Cys659Ter	2	1CH, 1GM	Masamune [11]

*N* number of patients. Number of patients according to nationality (Austrian [AU], Chinese [CH], French [FR], German [GM], Indian [IN], Japanese [JP], and Polish [PO]) is presented

^*^One patient was homozygous for p.Arg174Ter, and another for p.Arg646Trp

### TRPV6 activity was impaired in the presence of the novel nonsynonymous *TRPV6* variants

To assess the functional impact of the nonsynonymous *TRPV6* variants described above, we performed Ca^2+^ imaging assays. The nonsense variant c.1137C>A [p.Tyr379Ter] was excluded, because it was predicted to abolish the Ca^2+^ pore domain essential for TRPV6 function [16]. Compared with cells expressing wild-type TRPV6, HEK293 cells expressing TRPV6 variants showed a significantly reduced increase in intracellular Ca^2+^ concentration ([Ca^2+^]_i_) (Supplementary Fig. 1).

### Minigene assay

**Fig. 1:**
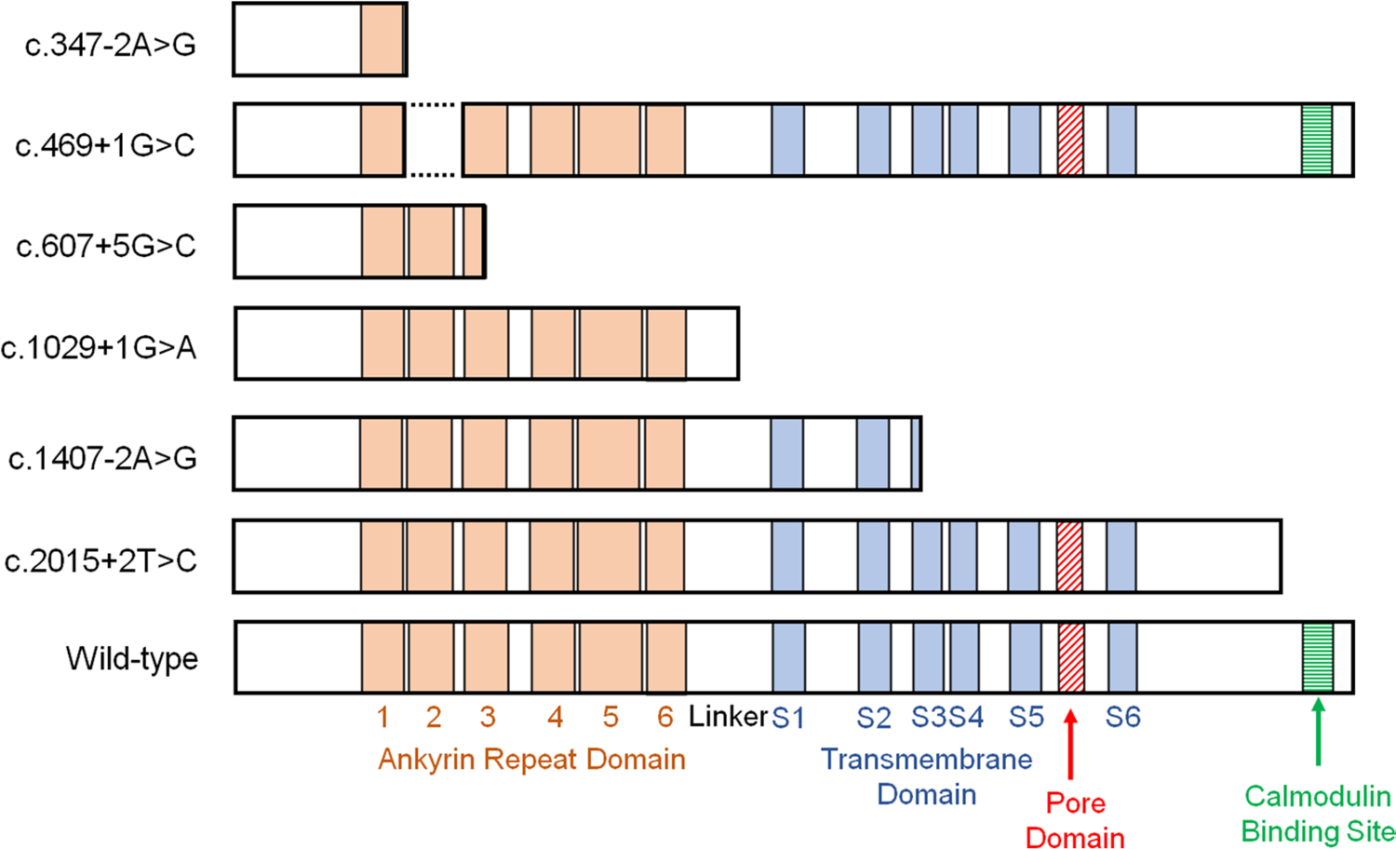
Predicted structures of the mutated TRPV6 protein based on the minigene assays. Normal splicing of the wild-type sequence and aberrant splicing of the mutant sequence are illustrated for each variant

We performed minigene assays to evaluate the splicing consequences of the splice-site variants (Table 2, Supplementary Figs. 2–7). Reverse transcription PCR of the wild-type construct generated a 457 bp fragment, whereas amplification of the c.347-2A>G construct yielded a larger 490 bp fragment due to retention of the first 33 bp of intron 2 before the exon 3 splice donor. This was predicted to result in the insertion of three new amino acids followed by a premature stop codon. The c.469+1G>C variant was predicted to cause skipping of the entire exon 3, resulting in the deletion of 41 amino acids and loss of the ANK2 domain. The c.607+5G>C variant was predicted to cause the skipping of the last 91 bp of exon 4, leading to a frameshift and early termination within the ANK3 domain. The c.1029+1G>A variant was predicted to include the entire intron 7, causing a frameshift and termination in the linker region. The c.1407-2A>G variant was predicted to cause complete exon 11 skipping, resulting in termination at amino acid 470. These five variants caused the loss of the Ca^2+^ pore domain. The c.2015 + 2T>C variant was predicted to include the first 17 bp of intron 14, generating 40 novel amino acids and the loss of the calmodulin binding site. Because loss of this site diminishes TRPV6 activity [11], the c.2015 + 2T>C variant was considered functionally impaired. Collectively, all six splice-site variants were predicted to impair TRPV6 function and to be pathogenic [35]. The predicted structural consequences based on the minigene assays are summarized in Fig. 1.

**Table 2:** Predicted splicing outcome of splice-site variants based on minigene assays

Splice site variants*	Location (boundary)	Splicing outcome: RNA*	Protein change**
c.347-2A>G	Intron 2/exon 3	r.346_347ins[347-33_347-3;gg]	p.(Gly116_Gln764delinsGluAla)
c.469+1G>C	Exon 3/intron 3	r.348_470del exon 3	p.(Ala117_Gly157del)
c.607+5G>C	Exon 4/intron 4	r.518_608del	p.(Val173Glyfs*11)
c.1029+1G>A	Exon 7/intron 7	r.1029_1030ins[a;1029+2_1030-1]	p.(Ala344Ilefs*36)
c.1407-2A>G	Intron 10/exon 11	r.1407_1572del	p.(Ile470*)
c.2015+2T>C	Exon 14/intron 14	r.2015_2016ins[gc;2015+3_2015+17]	p.(Val673Argfs*41)

^*^According to NM_018646.6

^*^^*^According to NP_061116.5

### Overview of the patients carrying functionally impaired *TRPV6* variants

We aimed to clarify the clinical characteristics of patients carrying functionally impaired *TRPV6* variants. A total of 45 nonsynonymous variants (41 previously reported and four identified in this study) and six splice-site variants were included. In total, 94 patients with alcohol-unrelated pancreatitis (70 with CP and 24 with RAP) were retrospectively analyzed (Table 3), comprising 33 French, 20 Japanese, 18 Chinese, 11 German, 7 Polish, 3 Indian, and 2 Austrian patients. These patients are collectively referred to as having *TRPV6*-related pancreatitis. Of the 94 patients, 54 (57.4%) were male. Thirty-two (34.0%) had a family history of pancreatitis and were classified as hereditary/familial, while 62 (66.0%) were classified as idiopathic. The median age at the final follow-up was 26 years. Two patients carried homozygous *TRPV6* variants (p.Arg174Ter and p.Arg646Trp, one each). Although no patients were compound heterozygous for functionally impaired *TRPV6* variants as defined herein, eight patients had other nonsynonymous *TRPV6* variants (p.Ala210Val/p.Asp324Asn, p.Ile223Thr/p.Leu392Phe, p.Leu392Phe/p.Gly451Glu, p.Ile223Thr/p.Arg425Gln, p.Ile223Thr/p.Gly428Arg, p.Ile223Thr/p.Ile580Phe, p.Val492Leu/p.Ala626Pro, and c.607+5G>C/p.Ile223Thr, all one each). Twenty-one (22.3%) patients were double heterozygous for pancreatitis risk variants in other susceptibility genes, including 14 with *SPINK1* (8 with p.Asn34Ser and 6 with c.194+2T>C), 2 with *CTRC* (both p.Arg254Trp), and 5 with *CFTR* (3 with p.Phe508del and 2 with p.Arg117His) [36]. No patients carried pathogenic *PRSS1* variants (p.Arg122His or p.Asn29Ile).

**Table 3:** Clinical characteristics of enrolled patients

	*TRPV6*-related (*n* = 94)	*PRSS1*-related (*n* = 68)	*SPINK1*-related (*n* = 90)	PV-negative* (*n* = 314)
Sex, male,* n* (%)	54 (57.4)	43 (63.2)	45 (50)	176 (56.1)
CP/RAP	70/24	54/14	68/22	234/80
Etiology, *n* (%)				
Idiopathic	62	6 (sporadic)	68	287
Hereditary/familial	32	62	22	22
Pancreas divisum	0	0	0	5
Median (95% CI) age at last follow-up	26 (22.4–29.7)	23 (14.0–32.0)	23 (17.1–28.9)	45 (40.3–49.7)
Onset of symptoms				
Yes, *n* (%)	89 (94.7)	67 (98.5)	85 (94.4)	270 (86.0)
Median (95% CI) age at pain onset, years	16 (13.6–18.4)	8 (4.0–12.0)	14 (11.5–16.5)	32 (27.1–36.9)
Pancreatic calcification				
Yes, *n* (%)	49 (52.1)	41 (60.3)	64 (71.1)	190 (61.5)**
Median (95% CI) age at diagnosis, years	29 (36.9–45.1)	27 (22.4–31.6)	23 (18.4–27.6)	54 (49.4–58.6)
Pancreatic exocrine insufficiency				
Yes, *n* (%)	21 (22.3)	31 (45.6)	25 (27.8)	84 (27.2)**
Median (95% CI) age at diagnosis, years	Not reached	35 (30.3–39.7)	51 (38.6–63.4)	72 (66.2–77.8)
Diabetes mellitus				
Yes, *n* (%)	15 (16.0)	19 (27.9)	19 (21.1)	85 (27.5)**
Median (95% CI) age at diagnosis, years	Not reached	42 (26.8–57.2)	58 (41.0–75.0)	72 (65.7–78.3)
Intervention for pancreatitis				
Yes, *n* (%)	37 (39.4)	29 (42.6)	48 (53.3)	131 (41.7)
Median (95% CI) age at diagnosis, years	35 (29.1–40.9)	33 (25.0–41.0)	32 (29.6–34.4)	63 (58.2–67.8)
Endoscopic treatment				
Yes, *n* (%)	33 (35.1)	17 (25)	44 (48.9)	124 (39.5)
Median (95% CI) age at the first treatment, years	37 (30.0–44.0)	Not reached	32 (23.5–40.5)	63 (58.7–67.3)
Surgery				
Yes, *n* (%)	7 (7.5)	16 (23.5)	12 (13.3)	32 (10.2)
Median (95% CI) age at pain onset, years	Not reached	Not reached	Not reached	Not reached
Pancreatic cancer				
Yes, *n* (%)	0 (0)	1 (1.5)	1 (1.1)	8 (2.5)
Median (95% CI) age at diagnosis, years	Not reached	Not reached	Not reached	Not reached

*CI* confidence intervals, *CP* chronic pancreatitis, *IQR* Interquartile range, *RAP* recurrent acute pancreatitis

^*^No pathogenic variants (PVs) were detected in *TRPV6*, *PRSS1*, *SPINK1*, *CTRC*, or *CPA1*

^**^Information about the presence or absence of the complication was not available in 6 patients

#### Onset of symptoms

**Fig. 2:**
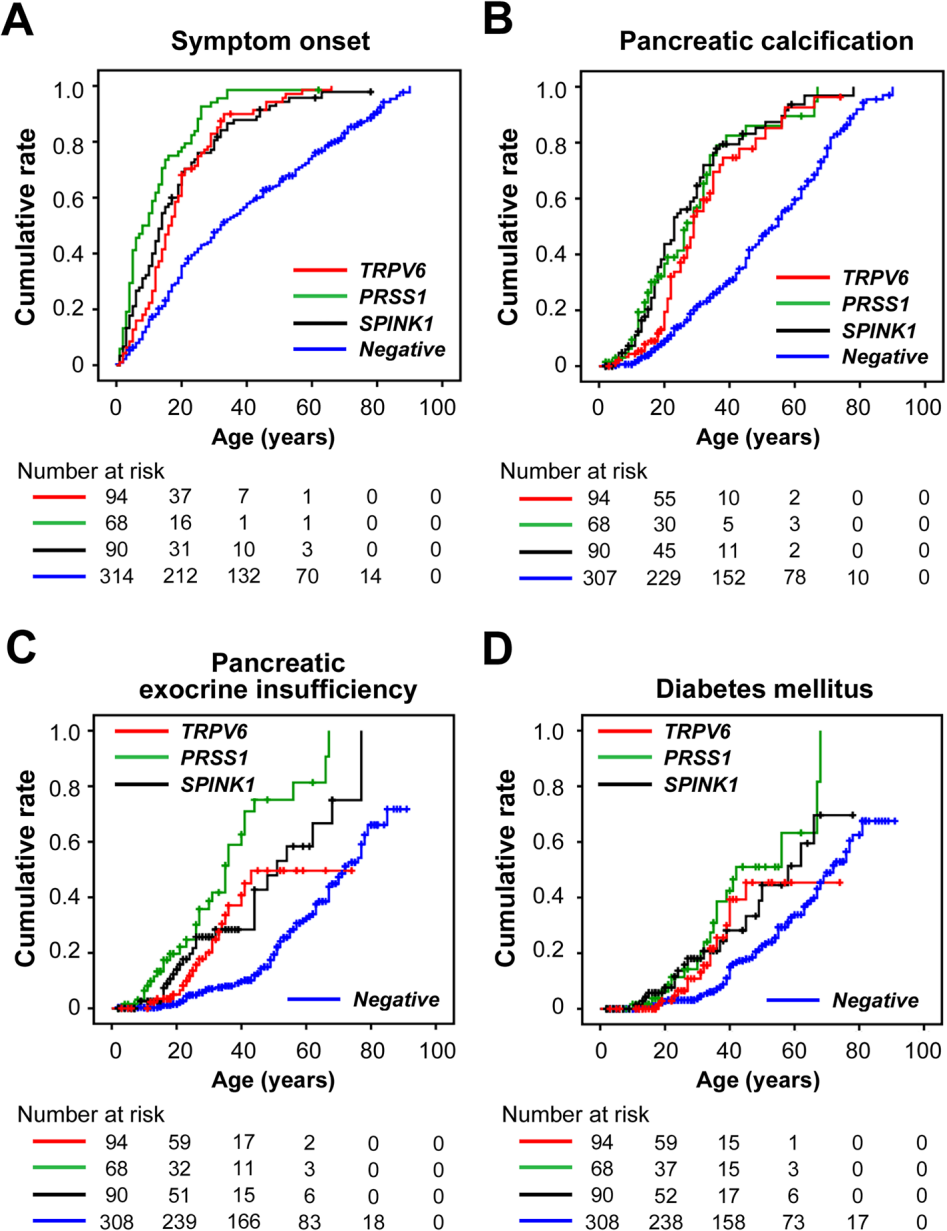
Comparison of different pathogenic genotypes on clinical outcomes. Kaplan–Meier curves showing the cumulative rates of **A** symptom onset, **B** pancreatic calcification, **C** pancreatic exocrine insufficiency, and **D** diabetes mellitus, according to genotype (*TRPV6*-related, *PRSS1*-related, *SPINK1*-related, or PV-negative pancreatitis). Censored subjects are indicated on the Kaplan–Meier curves by tick marks

Most patients with *TRPV6*-related pancreatitis developed symptoms by the age of 30 (Fig. 2A, Supplementary Fig. 8A). The median age at symptom onset was 16 years. Twelve patients (12.8%), 21 (22.3%), 64 (68.1%), and 76 (80.9%) developed symptoms by the ages of 5, 10, 20, and 30, respectively. Among the 89 symptomatic patients, the initial presentation was acute pancreatitis in 67 patients, abdominal pain in 14, abdominal and back pain in 6, and vomiting in 2 patients. We compared the age at symptom onset in patients with *TRPV6*-related pancreatitis to those with *PRSS1*-related pancreatitis (p.Arg122His and p.Asn29Ile; *n* = 68), *SPINK1*-related pancreatitis (p.Asn34Ser, p.P45Ser, and c.194+2T>C; *n* = 90) [37], and PV-negative pancreatitis (*n* = 314) (Table 3, Supplementary Table 8). The median age at symptom onset (95% CI) was 8 years (4.0–12.0) in *PRSS1*-related pancreatitis, 14 years (11.5–16.5) in *SPINK1*-related pancreatitis, and 32 years (27.1–36.9) in PV-negative pancreatitis. The age at symptom onset in *TRPV6*-related pancreatitis was significantly younger than that in PV-negative pancreatitis (*P* < 0.001) but older than that in *PRSS1*-related pancreatitis (*P* = 0.001). No significant difference was observed between *TRPV6*- and *SPINK1*-related pancreatitis.

#### Pancreatic calcification

Forty-nine patients (52.1%) with *TRPV6*-related pancreatitis were diagnosed with pancreatic calcification, with a median age of 29 years (Fig. 2B, Supplementary Fig. 8B). The cumulative incidence rates of pancreatic calcification were 19.4%, 55.5%, 74.6%, and 81.5% at the ages of 20, 30, 40, and 50 years, respectively. The age at diagnosis was significantly younger in patients with *TRPV6*-related pancreatitis than in those with PV-negative pancreatitis (*P* < 0.001). No significant differences were observed when compared with patients with *PRSS1*-related or *SPINK1*-related pancreatitis.

#### PEI and DM

Twenty-one patients (22.3%) and 15 patients (16.0%) with *TRPV6*-related pancreatitis were diagnosed with PEI and DM, respectively (Fig. 2C, D, Supplementary Fig. 8C, D). The cumulative rates of PEI were 4.9%, 20.1%, 40.8%, and 49.6%, and those of DM were 2.9%, 10.8%, 39.3%, and 45.4% at the ages of 20, 30, 40, and 50, respectively. The age at diagnosis of PEI and DM was significantly younger in patients with *TRPV6*-related pancreatitis compared to those with PV-negative pancreatitis (*P* < 0.001 for PEI and *P* = 0.006 for DM). In contrast, the age at diagnosis of PEI was older in *TRPV6*-related pancreatitis patients than in *PRSS1*-related pancreatitis (*P* = 0.002), while no significant difference was observed for DM. There was no significant difference in the age at diagnosis of either PEI or DM between patients with *TRPV6*-related and *SPINK1*-related pancreatitis.

#### Interventions

**Fig. 3:**
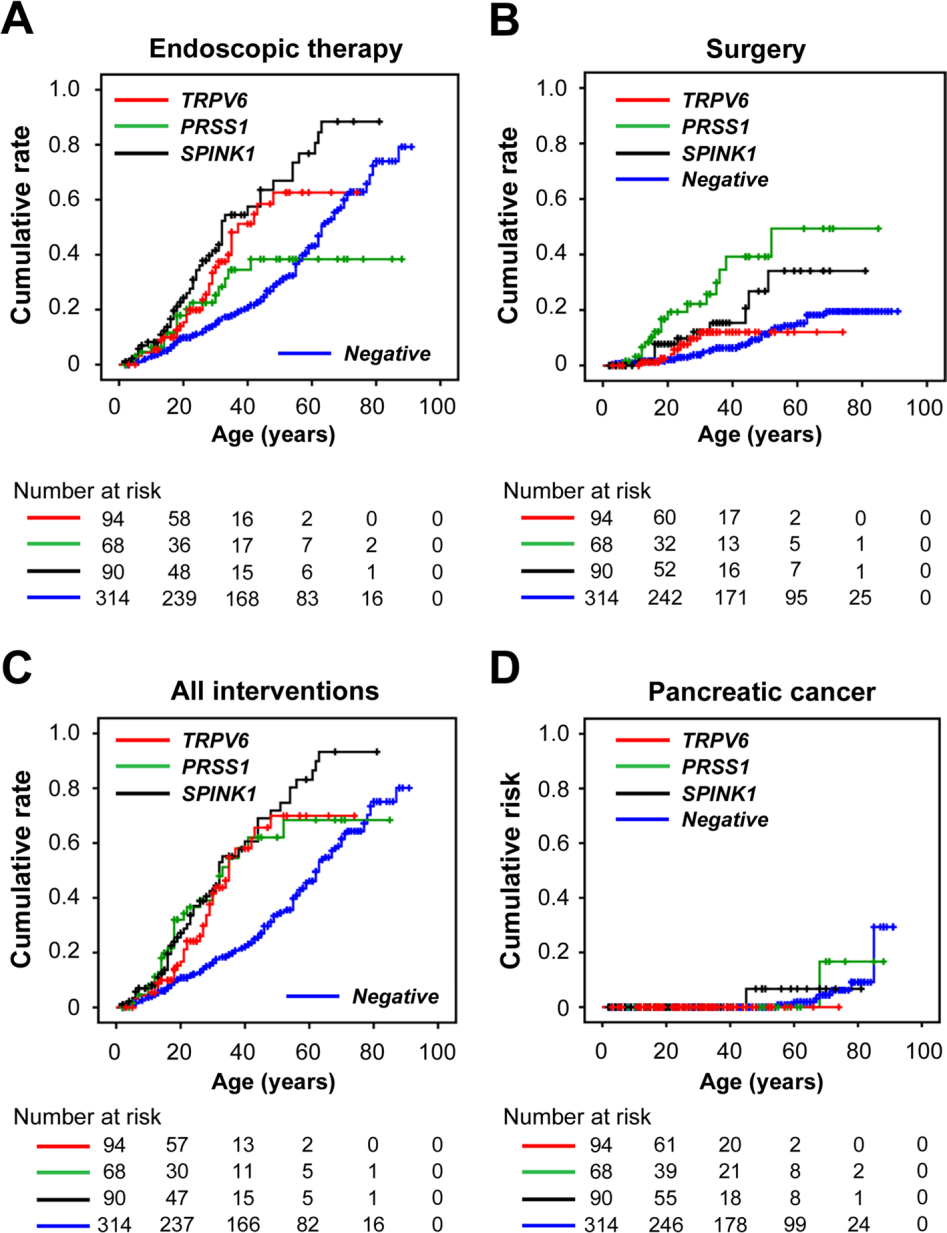
Timing of the first interventions for pancreatitis and the diagnosis of pancreatic cancer according to pathogenic genotypes. Kaplan–Meier curves showing the cumulative rates of **A** endoscopic treatment, **B** surgery, **C** all interventions, and **D** pancreatic cancer diagnosis according to genotype (*TRPV6*-related, *PRSS1*-related, *SPINK1*-related, or PV-negative pancreatitis). Censored subjects are indicated on the Kaplan–Meier curves by tick marks

Among the 94 patients with *TRPV6*-related pancreatitis, 37 (39.4%) underwent intervention: endoscopic treatment alone in 30 (81.1%), surgery alone in 4 (4.3%), a step-up approach in 2 (2.1%), and a top-down approach in 1 (1.1%). The median ages at first endoscopic treatment and at first intervention (either endoscopic or surgical) were 37 and 35 years, respectively (Fig. 3A–C, Supplementary Fig. 9A–C). The cumulative rates of all interventions were 16.7%, 41.6%, 58.0%, and 69.9% at ages 20, 30, 40, and 50 years, respectively. For endoscopic treatment, the cumulative rates were 15.4%, 35.4%, 51.2%, and 62.6% at ages 20, 30, 40, and 50 years, respectively. For surgery, the cumulative rates were 2.5% and 12.0% at ages 20 and 30 years, respectively. The age at first intervention was significantly younger in patients with *TRPV6*-related pancreatitis than in those with PV-negative pancreatitis (*P* < 0.001), whereas no significant differences were observed compared with patients with *PRSS1*- or *SPINK1*-related pancreatitis. Similar results were observed for endoscopic treatment (*P* < 0.001 vs. PV-negative, *P* = 0.21 vs. *PRSS1*, *P* = 0.12 vs. *SPINK1*). In contrast, patients with *TRPV6*-related pancreatitis underwent their first procedure at an older age than those with *PRSS1*-related pancreatitis (*P* = 0.002), whereas no significant differences were observed compared with patients with *SPINK1*-related or PV-negative pancreatitis.

#### Diagnosis of pancreatic cancer

Pancreatic cancer was not observed in patients with *TRPV6*-related pancreatitis. In contrast, it was diagnosed in one patient with the *PRSS1* p.Arg122His variant, one patient with the *SPINK1* p.Asn34Ser variant, and eight patients with PV-negative pancreatitis (Fig. 3D, Supplementary Fig. 9D).

### Subgroup analysis excluding double-heterozygous patients

Among the 94 patients with *TRPV6*-related pancreatitis, 14 were double heterozygous for *TRPV6* and *SPINK1* variants. Therefore, a subgroup analysis was conducted on the remaining 80 patients (Supplementary Tables 9 and 10; Supplementary Figs. 10 and 11). All significant differences observed in the overall cohort (*n* = 94) remained statistically significant in this subgroup. In this subgroup, the ages at diagnosis of pancreatic calcification (*P* = 0.03) and at first endoscopic treatment (*P* = 0.041) were significantly higher than those in patients with *SPINK1*-related pancreatitis.

### Caerulein-induced pancreatitis was exacerbated in pancreas-specific *Trpv6* knockout mice

Finally, we investigated whether deletion of *Trpv6* in the pancreas affects the progression of pancreatitis in mice. To this end, we established pancreas-specific *Trpv6* CKO mice (Supplementary Fig. 12). A shorter PCR band was detected in pancreatic genomic DNA of *Trpv6* CKO mice, indicating *Cre*-mediated recombination, whereas other organs (lung, heart, liver) showed no evidence of recombination. Neither the floxed nor CKO mice exhibited significant histological abnormalities in the pancreas up to 90 days of age, similar to the *Trpv6*^mut/mut^ mice [11]. No PanIN formation was detected in *Trpv6* CKO mice, even at 180 days of age (data not shown).

**Fig. 4:**
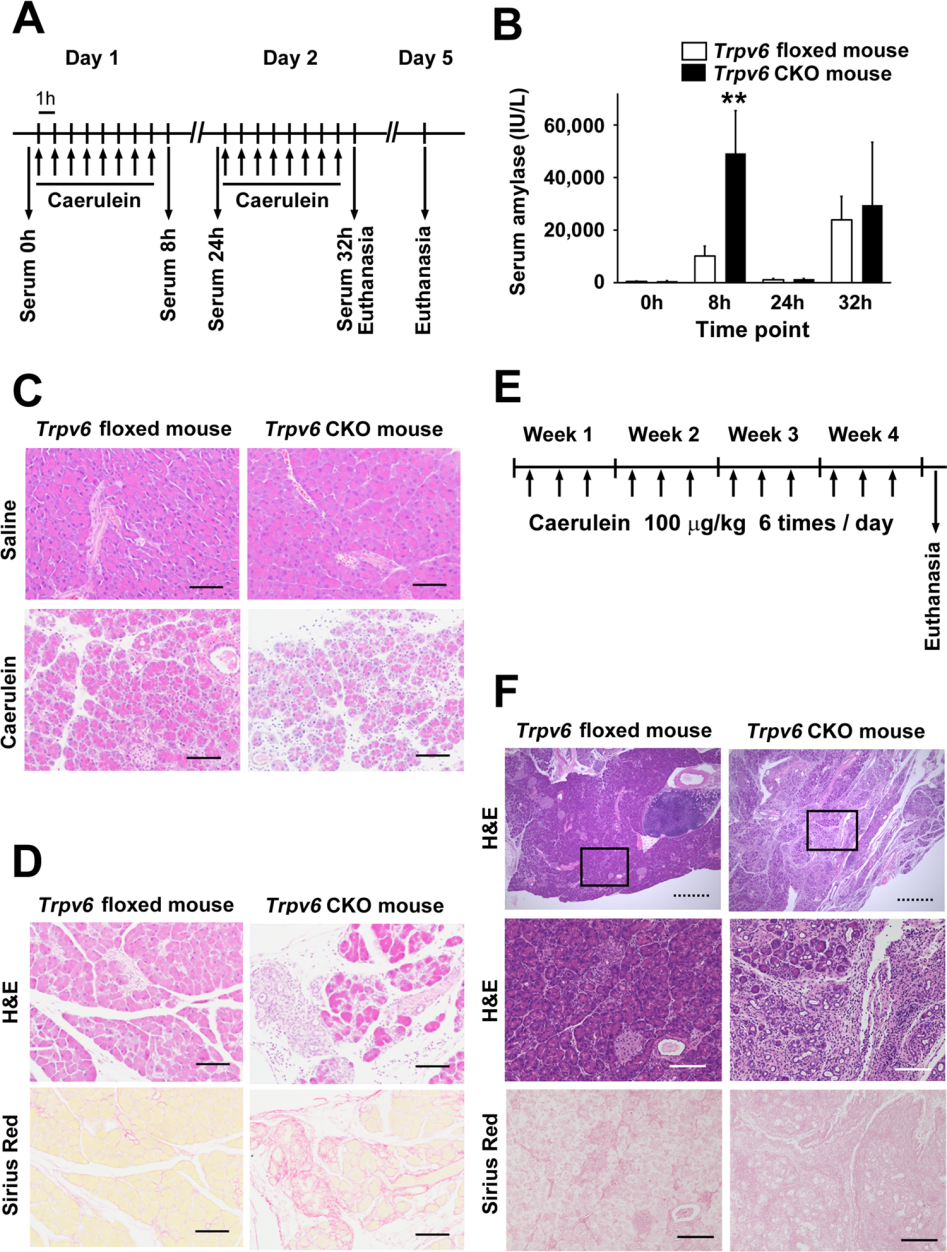
Caerulein-induced pancreatitis was exacerbated in pancreas-specific *Trpv6* conditional knockout mice. **A**–**D** *Trpv6* floxed mice and pancreas-specific *Trpv6* CKO mice received eight hourly intraperitoneal injections of caerulein (100 μg/kg body weight) or saline for two consecutive days. Blood samples were collected at 0 h (just before the first caerulein injection), 8 h, 24 h, and 32 h, and mice were euthanized at either 32 h or 96 h. **A** Scheme of the experiments. **B** Blood samples were obtained at the indicated time points, and serum amylase levels were measured. **C** Representative H&E staining of the pancreas at 32 h. Scale bar = 100 μm. **D** Representative H&E staining and Sirius Red staining of the pancreas at 96 h after the first caerulein injection. **E**, **F** CP was induced by 6-hourly intraperitoneal injections of caerulein (100 μg/kg body weight), 3 days/week for four consecutive weeks. **E** Scheme of the experiments. **F** Representative H&E staining and Sirius Red staining of the pancreas after 4 weeks

We induced pancreatitis by eight repetitive injections of caerulein over two consecutive days (Fig. 4A–D). Serum amylase levels increased 8 h after the first caerulein injection, decreased at 24 h, and elevated again at 32 h. Serum amylase levels were significantly higher in *Trpv6* CKO mice than in control floxed mice at 8 h. Histologically, *Trpv6* CKO mice developed more severe pancreatitis, as shown by more severe pancreatic edema, inflammatory cell infiltration, and acinar necrosis, compared to *Trpv6* floxed mice (Supplementary Fig. 13). On day 5, pancreatic fibrosis was more evident in *Trpv6* CKO mice, as assessed by Sirius Red staining.

CP was induced by six intraperitoneal injections of caerulein, administered 3 days/week for four consecutive weeks (Fig. 4E). Histological analysis revealed that *Trpv6* CKO mice exhibited more severe pancreatitis than floxed controls, characterized by increased inflammatory cell infiltration, acinar cell loss, and fibrosis (Fig. 4F). These findings indicate that pancreatic *Trpv6* plays a protective role in both acute and chronic pancreatitis in mice.

### Effects of *Trpv6* deletion on pancreatic organoids

*TRPV6*-related pancreatitis has been proposed as a “channelopathy” that impairs ductal secretion [11, 38], based on its predominant expression in human pancreatic duct cells identified by single-cell transcriptome profiling [39]. To investigate the effect of *Trpv6* deletion on ductal cells, we generated pancreatic organoids from *Trpv6* floxed and *Trpv6* CKO mice (Supplementary Fig. 14). These organoids expressed ductal markers such as *Slc9a1* and *Slc4a4*, whereas acinar markers such as *Prss1* and *Spink1* were undetectable, confirming their ductal phenotype.

Treatment with forskolin increased organoid size, indicating enhanced fluid secretion into the lumen [34]. However, the relative increase in organoid size after forskolin stimulation did not differ significantly between *Trpv6* floxed and *Trpv6* CKO organoids. Similarly, both groups showed comparable growth and morphological responses following Ca^2+^ treatment at various concentrations. These findings suggest that *Trpv6* deletion does not significantly affect the function or phenotype of pancreatic ductal organoids.

### TRPV6 expression is upregulated in pancreatic acinar cells in response to caerulein treatment

We hypothesized that TRPV6 is upregulated in pancreatic acinar cells following pancreatitis stimuli and may play a protective role against pancreatitis. Immunohistochemical staining revealed increased TRPV6 expression at 32 h after caerulein treatment in *Trpv6* floxed mice, whereas no such increase was observed in *Trpv6* CKO mice (Supplementary Fig. 15). In addition, repeated intraperitoneal injections of caerulein elevated *Trpv6* mRNA expression in the pancreas of male C57BL/6J mice. These findings indicate that TRPV6 is upregulated in pancreatic acinar cells in response to caerulein stimulation.

## Discussion

In this study, we aimed to address two unresolved questions regarding *TRPV6*-related pancreatitis: its natural history and the role of pancreas-specific *Trpv6* in pancreatitis in mice. Ninety-four patients with functionally impaired *TRPV6* variants, including 45 nonsynonymous and six splice-site variants, were enrolled. We demonstrated that pathogenic *TRPV6* variants influence the clinical course of pancreatitis, including earlier symptom onset and an increased risk of complications such as pancreatic calcification, PEI, and DM. In addition, we showed that caerulein-induced acute and chronic pancreatitis was more severe in pancreas-specific *Trpv6* CKO mice compared with the control floxed mice. Collectively, our results indicate that pancreatic TRPV6 plays a protective role against pancreatitis in both humans and mice.

Geographical and ethnic differences influence the impacts of environmental and genetic risk factors on pancreatitis. Alcohol-related CP is the leading cause of CP in Western countries and Japan [2–4], whereas idiopathic CP predominates in China, accounting for nearly three-quarters of cases [40]. Regarding genetic susceptibility, the *SPINK1* c.194+2T>C variant is relatively common in individuals of East Asian ancestry. A meta-analysis reported an odds ratio (OR) of 25.73 for CP in East Asians carrying this variant, compared with 10.21 in non-East Asians [41]. Its effect is particularly pronounced in idiopathic CP (OR 35.31) compared to non-idiopathic CP (OR 25.75). In contrast, the *SPINK1* p.Asn34Ser variant is more frequently observed in individuals of European descent, with an OR of 9.70 [42]. Its association is stronger in idiopathic CP (OR 13.6) than in alcohol-related CP (OR 5.3).

Previous studies have shown the natural history of *PRSS1*-related and *SPINK1*-related pancreatitis. Rebours et al. [43] reported that the median ages at disease onset, PEI diagnosis, and DM diagnosis in patients with *PRSS1*-related hereditary pancreatitis were 10, 30, and 37 years, respectively. A nationwide epidemiological survey of hereditary pancreatitis in Japan showed that the mean age at symptom onset was 12.3 years for patients with *PRSS1*-related hereditary pancreatitis and 20 years for those with *SPINK1*-related cases [44]. The median ages at diagnosis of PEI and DM were 35 and 42 years, respectively, for patients with *PRSS1*-related hereditary pancreatitis and 42 and 61 years for those with *SPINK1*-related cases. The median ages at symptom onset, diagnosis of pancreatic stones, and diagnosis of DM were 44.2 years, 51.0 years, and 67.0 years, respectively, in PV-negative patients with idiopathic CP in China [45]. Muller et al. [24] reported the natural history of 209 patients with *SPINK1*-related pancreatitis. The median ages at symptom onset and at the diagnosis of PEI were 20.1 years and 49.5 years, respectively. The clinical outcomes of the control cohorts in our study were similar to those reported in previous studies. Our results demonstrated that PVs in *TRPV6* significantly influenced the age of symptom onset and clinical outcomes in CP, albeit to a lesser extent than PVs in *PRSS1*.

In addition to nonsynonymous variants, we analyzed splice-site *TRPV6* variants. While a previous study identified the c.607+5G>A splice-site variant in a child with transient neonatal hyperparathyroidism [46], no prior studies have explored the role of splice-site *TRPV6* variants in pancreatitis. The c.607+5G>A variant caused the insertion of the first 41 bp of intron 4, resulting in a frameshift and termination at the ANK repeat 4. We identified another splice-site variant at the same position c.607+5G>C variant, which was predicted to cause partial deletion of exon four according to a minigene assay. The protein translated from the mutant mRNA is predicted to be truncated, containing a Val173Gly substitution, followed by a novel 9-amino-acid sequence and premature termination. These results suggest the diversity of the impact of the splice-site variants on the splicing machinery of the *TRPV6* gene. Interestingly, full-gene splicing assays have revealed the pathogenic roles of intronic, non-splice-site variants in other pancreatitis susceptibility genes, such as *SPINK1* [47]. Further studies are needed to clarify the pathogenic role of other non-canonical intronic *TRPV6* variants in pancreatitis.

We showed that pancreatic *Trpv6* is essential for protection against pancreatitis in mice. Since *TRPV6*-related pancreatitis has been proposed as a “channelopathy” that impairs ductal secretion [11, 38], we focused on ductal cells and assessed the impact of *Trpv6* deletion using pancreatic organoids. However, we observed no significant effects of *Trpv6* deletion on pancreatic organoids. In contrast, TRPV6 expression was markedly increased in pancreatic acinar cells in response to caerulein treatment, suggesting that its upregulation may protect against pancreatitis. Indeed, previous studies demonstrated significant TRPV6 expression in acinar cells in mice [48, 49]. Immunohistochemical analyses localized TRPV6 to exocrine acinar cells, with signals predominantly observed in granular structures at the apical membrane [49]. Similarly, fluorescent labeling in *Trpv6* reporter animals, validated by mass spectrometry, confirmed strong expression in acinar cells [48]. Although the precise role of TRPV6 in the pancreas is not fully understood, it may be involved in removing excess Ca^2+^ by ductal cells, thus preventing the premature activation of digestive enzymes after the release of secretory cargo. Furthermore, TRPV6 may play a role in the reuptake of Ca^2+^ to replenish stores and secretory granules in acinar cells [16, 49]. Although beyond the scope of the present study, further investigations—such as analyses of Ca^2+^ signaling and trypsin activation in acinar cells isolated from *Trpv6* CKO mice—will be essential to elucidate the precise mechanisms underlying *TRPV6*-related pancreatitis.

Interestingly, recent studies have shown that TRPV6 mediates alcohol-induced gut barrier dysfunction, microbiota dysbiosis, endotoxemia, systemic inflammation, and liver injury [50, 51]. *Trpv6* deficiency or inhibition attenuates alcohol-induced Ca^2+^ influx, tight junction disruption, and barrier dysfunction. Individuals carrying functionally impaired *TRPV6* variants are likely susceptible to pancreatitis but might be resistant to alcohol-related liver injury. In our previous study, the *TRPV6* p.Ile223Thr variant was overrepresented in alcohol-related CP [11]. It would be of interest to see whether the *TRPV6* variants are associated with alcohol-related liver injury and whether they have an impact on organ-specific alcohol-related injury.

This study has several limitations. First, it was a retrospective multicenter study, which may have introduced variability due to differences in diagnostic criteria and data collection methods. Although the fundamental concepts are similar, no international consensus criteria for CP currently exist. Therefore, we also included patients with RAP to avoid excluding cases that failed to meet the diagnostic criteria for CP. Complications may have developed before diagnosis if patients did not undergo regular examinations. Second, our control group consisted exclusively of Japanese patients, which may limit comparability with international cohorts. Ethnically diverse control cohorts with comparable clinical and genetic data were not available for this study. Nonetheless, the clinical outcomes of our control patients were generally consistent with those reported in previous studies [24, 43–45]. Third, patients carrying PVs in pancreatitis susceptibility genes did not have a long follow-up period. This may partly explain the low incidence of pancreatic cancer in patients with *PRSS1*-related pancreatitis, who are known to be at high risk for pancreatic cancer [52]. Fourth, we defined the functional impairment of *TRPV6* variants using Ca^2+^ imaging and minigene assays. Presumed gain-of-function variants, such as p.Gly451Ala and p.Gly451Glu, were not included. We were unable to perform RNA analysis on patient samples due to their unavailability. Lastly, the impact of *Trpv6* deletion was evaluated only using the caerulein-induced pancreatitis model. Despite these limitations, this is the first study to clarify the clinical outcomes of *TRPV6*-related pancreatitis in humans and the impact of pancreas-specific *Trpv6* deletion on pancreatitis in mice.

## Conclusions

Functionally impaired *TRPV6* variants influence the clinical outcomes of pancreatitis. Mice lacking pancreatic *Trpv6* are phenotypically normal but develop more severe acute and chronic pancreatitis in response to caerulein treatment. These findings indicate the essential role of TRPV6 in pancreatic health. Further studies are needed to clarify the pathogenic mechanism of *TRPV6*-related pancreatitis and to develop specific treatments targeting TRPV6.

## Supplementary Information

Below is the link to the electronic supplementary material.

Supplementary file1 (DOCX 37 KB)

Supplementary file2 (DOCX 36200 KB)

## Abbreviations

CI: Confidence intervals
CKO: Conditional knockout
CP: Chronic pancreatitis
DM: Diabetes mellitus
Fura-2-AM: Fura-2-acetoxymethyl ester
H&E: Hematoxylin and eosin
OR: Odds ratio
PEI: Pancreatic exocrine insufficiency
PV: Pathogenic variant
RAP: Recurrent acute pancreatitis
TRPV6: Transient receptor potential vanilloid subfamily member 6

## References

[1] Whitcomb DC, Frulloni L, Garg P, et al. Chronic pancreatitis: an international draft consensus proposal for a new mechanistic definition. Pancreatology. 2016;16:218–24. 26924663 10.1016/j.pan.2016.02.001 PMC6042966

[2] Mayerle J, Sendler M, Hegyi E, et al. Genetics, cell biology, and pathophysiology of pancreatitis. Gastroenterology. 2019;156:1951-68.e1. 30660731 10.1053/j.gastro.2018.11.081 PMC6903413

[3] Beyer G, Habtezion A, Werner J, et al. Chronic pancreatitis. Lancet. 2020;396:499–512. 32798493 10.1016/S0140-6736(20)31318-0

[4] Hines OJ, Pandol SJ. Management of chronic pancreatitis. BMJ. 2024;384:e070920. 38408777 10.1136/bmj-2023-070920

[5] Whitcomb DC, Gorry MC, Preston RA, et al. Hereditary pancreatitis is caused by a mutation in the cationic trypsinogen gene. Nat Genet. 1996;14:141–5. 8841182 10.1038/ng1096-141

[6] Sharer N, Schwarz M, Malone G, et al. Mutations of the cystic fibrosis gene in patients with chronic pancreatitis. N Engl J Med. 1998;339:645–52. 9725921 10.1056/NEJM199809033391001

[7] Cohn JA, Friedman KJ, Noone PG, et al. Relation between mutations of the cystic fibrosis gene and idiopathic pancreatitis. N Engl J Med. 1998;339:653–8. 9725922 10.1056/NEJM199809033391002

[8] Witt H, Luck W, Hennies HC, et al. Mutations in the gene encoding the serine protease inhibitor, Kazal type 1 are associated with chronic pancreatitis. Nat Genet. 2000;25:213–6. 10835640 10.1038/76088

[9] Rosendahl J, Witt H, Szmola R, et al. Chymotrypsin C (CTRC) variants that diminish activity or secretion are associated with chronic pancreatitis. Nat Genet. 2008;40:78–82. 18059268 10.1038/ng.2007.44 PMC2650829

[10] Witt H, Beer S, Rosendahl J, et al. Variants in CPA1 are strongly associated with early onset chronic pancreatitis. Nat Genet. 2013;45:1216–20. 23955596 10.1038/ng.2730 PMC3909499

[11] Masamune A, Kotani H, Sörgel FL, et al. Variants that affect function of calcium channel TRPV6 are associated with early-onset chronic pancreatitis. Gastroenterology. 2020;158:1626-41.e8. 31930989 10.1053/j.gastro.2020.01.005

[12] Zou WB, Wang YC, Ren XL, et al. TRPV6 variants confer susceptibility to chronic pancreatitis in the Chinese population. Hum Mutat. 2020;41:1351–7. 32383311 10.1002/humu.24032

[13] Hamada S, Masson E, Chen JM, et al. Functionally deficient *TRPV6* variants contribute to hereditary and familial chronic pancreatitis. Hum Mutat. 2022;43:228–39. 34923708 10.1002/humu.24315

[14] Oracz G, Zaród M, Ewers M, et al. Loss of function TRPV6 variants are associated with chronic pancreatitis in nonalcoholic early-onset Polish and German patients. Pancreatology. 2021;21:1434–42. 34538581 10.1016/j.pan.2021.09.005

[15] Shah IA, Prasad H, Banerjee S, et al. A novel frameshift mutation in TRPV6 is associated with hereditary pancreatitis. Front Genet. 2023;13:1058057. 36699452 10.3389/fgene.2022.1058057 PMC9868559

[16] Khattar V, Wang L, Peng JB. Calcium selective channel TRPV6: structure, function, and implications in health and disease. Gene. 2022;817:146192. 35031425 10.1016/j.gene.2022.146192 PMC8950124

[17] Fecher-Trost C, Wissenbach U, Weissgerber P. TRPV6: from identification to function. Cell Calcium. 2017;67:116–22. 28501141 10.1016/j.ceca.2017.04.006

[18] Bianco SD, Peng JB, Takanaga H, et al. Marked disturbance of calcium homeostasis in mice with targeted disruption of the *Trpv6* calcium channel gene. J Bone Miner Res. 2007;22:274–85. 17129178 10.1359/jbmr.061110 PMC4548943

[19] Weissgerber P, Kriebs U, Tsvilovskyy V, et al. Male fertility depends on Ca^2+^ absorption by TRPV6 in epididymal epithelia. Sci Signal. 2011;4:ra27. 21540454 10.1126/scisignal.2001791

[20] Nakano E, Masamune A, Niihori T, et al. Targeted next-generation sequencing effectively analyzed the cystic fibrosis transmembrane conductance regulator gene in pancreatitis. Dig Dis Sci. 2015;60:1297–307. 25492507 10.1007/s10620-014-3476-9

[21] Sudo Y, Matsuo K, Tetsuo T, et al. Derived (mutated)-types of TRPV6 channels elicit greater Ca^2+^ influx into the cells than ancestral-types of TRPV6: evidence from *Xenopus* oocytes and mammalian cell expression system. J Pharmacol Sci. 2010;114:281–91. 20948163 10.1254/jphs.10169fp

[22] Masamune A, Kikuta K, Kume K, et al. Nationwide epidemiological survey of chronic pancreatitis in Japan: introduction and validation of the new Japanese diagnostic criteria 2019. J Gastroenterol. 2020;55:1062–71. 32676800 10.1007/s00535-020-01704-9

[23] Tandon RK, Sato N, Garg PK. Chronic pancreatitis: Asia-Pacific consensus report. J Gastroenterol Hepatol. 2002;17:508–18. 11982735 10.1046/j.1440-1746.2002.02762.x

[24] Muller N, Sarantitis I, Rouanet M, et al. Natural history of *SPINK1* germline mutation related-pancreatitis. EBioMedicine. 2019;48:581–91. 31628023 10.1016/j.ebiom.2019.09.032 PMC6838417

[25] Ru N, Xu XN, Cao Y, et al. The impacts of genetic and environmental factors on the progression of chronic pancreatitis. Clin Gastroenterol Hepatol. 2022;20:e1378–87. 34461303 10.1016/j.cgh.2021.08.033

[26] American Diabetes Association. 2. Classification and diagnosis of diabetes: standards of medical care in diabetes-2018. Diabetes Care. 2018;2018(41):S13-27. 10.2337/dc18-S002 29222373

[27] Jensen JN, Cameron E, Garay MV, et al. Recapitulation of elements of embryonic development in adult mouse pancreatic regeneration. Gastroenterology. 2005;128:728–41. 15765408 10.1053/j.gastro.2004.12.008

[28] Kikuta K, Masamune A, Hamada S, et al. Pancreatic stellate cells reduce insulin expression and induce apoptosis in pancreatic beta-cells. Biochem Biophys Res Commun. 2013;433:292–7. 23500461 10.1016/j.bbrc.2013.02.095

[29] Moreno C, Nicaise C, Gustot T, et al. Chemokine receptor CCR5 deficiency exacerbates caerulein-induced acute pancreatitis in mice. Am J Physiol Gastrointest Liver Physiol. 2006;291:G1089–99. 16891300 10.1152/ajpgi.00571.2005

[30] Matsumoto R, Hamada S, Tanaka Y, et al. Nuclear factor erythroid 2-related factor 2 depletion sensitizes pancreatic cancer cells to gemcitabine via aldehyde dehydrogenase 3a1 repression. J Pharmacol Exp Ther. 2021;379:33–40. 34321315 10.1124/jpet.121.000744

[31] Wang MJ, Wang YC, Masson E, et al. SEC16A variants predispose to chronic pancreatitis by impairing ER-to-Golgi transport and inducing ER stress. Adv Sci (Weinh). 2024;11:e2402550. 39119875 10.1002/advs.202402550 PMC11481239

[32] Broutier L, Andersson-Rolf A, Hindley CJ, et al. Culture and establishment of self-renewing human and mouse adult liver and pancreas 3D organoids and their genetic manipulation. Nat Protoc. 2016;11:1724–43. 27560176 10.1038/nprot.2016.097

[33] Ootani A, Li X, Sangiorgi E, et al. Sustained in vitro intestinal epithelial culture within a Wnt-dependent stem cell niche. Nat Med. 2009;15:701–6. 19398967 10.1038/nm.1951 PMC2919216

[34] Dekkers JF, Wiegerinck CL, de Jonge HR, et al. A functional CFTR assay using primary cystic fibrosis intestinal organoids. Nat Med. 2013;19:939–45. 23727931 10.1038/nm.3201

[35] Richards S, Aziz N, Bale S, et al. Standards and guidelines for the interpretation of sequence variants: a joint consensus recommendation of the American College of Medical Genetics and Genomics and the Association for Molecular Pathology. Genet Med. 2015;17:405–24. 25741868 10.1038/gim.2015.30 PMC4544753

[36] Berke G, Gede N, Szadai L, et al. Bicarbonate defective CFTR variants increase risk for chronic pancreatitis: a meta-analysis. PLoS ONE. 2022;17:e0276397. 36264955 10.1371/journal.pone.0276397 PMC9584382

[37] Kume K, Masamune A, Ariga H, et al. Do genetic variants in the SPINK1 gene affect the level of serum PSTI? J Gastroenterol. 2012;47:1267–74. 22526274 10.1007/s00535-012-0590-3

[38] Sahin-Tóth M. Channelopathy of the pancreas causes chronic pancreatitis. Gastroenterology. 2020;158:1538–40. 32205170 10.1053/j.gastro.2020.03.027 PMC7751598

[39] Segerstolpe Å, Palasantza A, Eliasson P, et al. Single-cell transcriptome profiling of human pancreatic islets in health and type 2 diabetes. Cell Metab. 2016;24:593–607. 27667667 10.1016/j.cmet.2016.08.020 PMC5069352

[40] Hao L, Wang LS, Liu Y, et al. The different course of alcoholic and idiopathic chronic pancreatitis: a long-term study of 2,037 patients. PLoS ONE. 2018;13:e0198365. 29883461 10.1371/journal.pone.0198365 PMC5993321

[41] Tang XY, Zou WB, Yu FF, et al. Meta-analysis of the impact of the SPINK1 c.194 + 2T > C variant in chronic pancreatitis. Dig Liver Dis. 2020;52:143–8. 31401021 10.1016/j.dld.2019.07.004

[42] Di Leo M, Bianco M, Zuppardo RA, et al. Meta-analysis of the impact of SPINK1 p. N34S gene variation in Caucasic patients with chronic pancreatitis. An update. Dig Liver Dis. 2017;49:847–53. 28546062 10.1016/j.dld.2017.04.023

[43] Rebours V, Boutron-Ruault MC, Schnee M, et al. The natural history of hereditary pancreatitis: a national series. Gut. 2009;58:97–103. 18755888 10.1136/gut.2008.149179

[44] Masamune A, Kikuta K, Hamada S, et al. Nationwide survey of hereditary pancreatitis in Japan. J Gastroenterol. 2018;53:152–60. 28861620 10.1007/s00535-017-1388-0

[45] Zou WB, Tang XY, Zhou DZ, et al. *SPINK1*, *PRSS1*, *CTRC*, and *CFTR* genotypes influence disease onset and clinical outcomes in chronic pancreatitis. Clin Transl Gastroenterol. 2018;9:204. 30420730 10.1038/s41424-018-0069-5 PMC6232107

[46] Suzuki Y, Chitayat D, Sawada H, et al. TRPV6 variants interfere with maternal-fetal calcium transport through the placenta and cause transient neonatal hyperparathyroidism. Am J Hum Genet. 2018;102:1104–14. 29861107 10.1016/j.ajhg.2018.04.006 PMC5992228

[47] Zou WB, Boulling A, Masson E, et al. Clarifying the clinical relevance of SPINK1 intronic variants in chronic pancreatitis. Gut. 2016;65:884–6. 26719302 10.1136/gutjnl-2015-311168

[48] Wartenberg P, Lux F, Busch K, et al. A TRPV6 expression atlas for the mouse. Cell Calcium. 2021;100:102481. 34628109 10.1016/j.ceca.2021.102481

[49] Zhuang L, Peng JB, Tou L, et al. Calcium-selective ion channel, CaT1, is apically localized in gastrointestinal tract epithelia and is aberrantly expressed in human malignancies. Lab Investig. 2002;82:1755–64. 12480925 10.1097/01.lab.0000043910.41414.e7

[50] Meena AS, Shukla PK, Bell B, et al. TRPV6 channel mediates alcohol-induced gut barrier dysfunction and systemic response. Cell Rep. 2022;39:110937. 35705057 10.1016/j.celrep.2022.110937 PMC9250449

[51] Hou Z, Ding Q, Li Y, et al. Intestinal epithelial β Klotho is a critical protective factor in alcohol-induced intestinal barrier dysfunction and liver injury. EBioMedicine. 2022;82:104181. 35908416 10.1016/j.ebiom.2022.104181 PMC9352463

[52] Howes N, Lerch MM, Greenhalf W, et al. Clinical and genetic characteristics of hereditary pancreatitis in Europe. Clin Gastroenterol Hepatol. 2004;2:252–61. 15017610 10.1016/s1542-3565(04)00013-8

