# Supplementary material for: TRPV6-related pancreatitis: natural history and the impact of the pancreas-specific deletion on pancreatitis in mice
Source: J Gastroenterol. 2025 Nov 17;61(2):207–21. doi: 10.1007/s00535-025-02323-y (PMC12924865; doi:10.1007/s00535-025-02323-y)
Supplement: Supplementary file 1 — Supplementary file1 (DOCX 37 KB) [file 535_2025_2323_MOESM1_ESM.docx]

| **Supplementary Table 1. Primer sequences used to amplify the flanking exons** | | | | |
| --- | --- | --- | --- | --- |
| Variant | Location of boundary | Exons amplified by PCR | Forward primer | Reverse primer |
| c.347-2A>G | intron 2/exon 3 | 2–5 | 5’-gaattcgatctgggagtctcctctccttctagct-3' | 5’-ctcgagttacagggagtcctgggcccggatgtca-3' |
| c.469+1G>C | exon 3/intron 3 | 2–5 | 5’-gaattcgatctgggagtctcctctccttctagct-3' | 5’-ctcgagttacagggagtcctgggcccggatgtca-3' |
| c.607+5G>C | exon 4/intron 4 | 4–7 | 5’-gaattccagactgcactgcacatcgctg-3' | 5’-ctcgagttactcccgcttcttggtggtgatg-3' |
| c.1029+1G>A | exon 7/intron 7 | 7–11 | 5’-gaattcatgtttcagcacctgatgcagaagcgga-3' | 5’-ctcgagttacttctgaatcatgatggtgaaggggc-3' |
| c.1407-2A>G | intron 10/exon 11 | 10–13 | 5’-gaattcgttccagacatcttcagaatgggggtca-3’ | 5’-ctcgagttaggccctccacagctcatcccgctcat-3’ |
| c.2015+2T>C | exon 14/intron 14 | 14–15 | 5’-gaattcgttgtggccaccacggtgatgct-3' | 5’-ctcgagagtcagatctgatattcccagctctcc-3' |

| **Supplementary Table 2. PCR primer sequences for the *in vitro* splicing assay** | | |
| --- | --- | --- |
| Variant | Forward primer | Reverse primer |
| c.347-2A>G | 5’-gatctgggagtctcctctccttctagct-3' | 5’-cagggagtcctgggcccggatgtca-3' |
| c.469+1G>C | 5’-gatctgggagtctcctctccttctagct-3' | 5’-cagggagtcctgggcccggatgtca-3' |
| c.607+5G>C | 5’-cagactgcactgcacatcgctg-3' | 5’-ctcccgcttcttggtggtgatg-3' |
| c.1029+1G>A | 5’-atgtttcagcacctgatgcagaagcgga-3' | 5’-cttctgaatcatgatggtgaaggggc-3' |
| c.1407-2A>G | 5’-gttccagacatcttcagaatgggggtca-3’ | 5’-ggccctccacagctcatcccgctcat-3’ |
| c.2015+2T>C | 5’-gttgtggccaccacggtgatgct-3' | 5’-tcagatctgatattcccagctctcc-3' |

| **Supplementary Table 3. Diagnostic criteria for CP and PEI used at the respective institutions** | | | |
| --- | --- | --- | --- |
| Country | Diagnostic criteria for CP | Diagnostic criteria for PEI | References |
| Japan | CP was diagnosed based on the clinical diagnostic criteria for CP 2019 proposed by the Japan Pancreas Society. Definite CP was diagnosed by the imaging criteria. Patients with probable CP findings plus two or more of the following—(i) recurrent upper abdominal or back pain, (ii) abnormal pancreatic enzyme levels in serum or urine, and (iii) impaired pancreatic exocrine function—were also classified as having definite CP. | PEI was diagnosed in the presence of clinical steatorrhea, a requirement for long-term oral pancreatic enzyme supplementation, and/or abnormal results on a BT-PABA test. | Masamune A, et al.  J Gastroenterol 2020;55: 1062-71 |
| France | CP was diagnosed if any of the following were present: pancreatic calcifications detected by CT or EUS; moderate to severe pancreatic ductal abnormalities on MRI with MRCP; or evidence of PEI. | PEI was diagnosed if any of the following were present: clinical steatorrhea, a fecal elastase-1 concentration <100 μg/g stool, or a requirement for long-term oral pancreatic enzyme supplementation. | Muller N, et al. EBioMedicine 2019:48: 581-591 |
| China | CP was diagnosed if any of the following were present: (i) characteristic histological features of CP in pancreatic tissue; (ii) pancreatic calcifications confirmed by CT, MRI, or EUS; (iii) moderate-to-severe pancreatic ductal abnormalities on ERCP or MRCP; or (iv) abnormal pancreatic function test results. | PEI was diagnosed if either of the following was present: chronic diarrhea with foul-smelling, oily stools, or a positive result on a standard quantitative test (fecal fat excretion >14 g/day). | Tandon RK, et al.  J Gastroenterol Hepatol 2002;17:508-18.  Ru N, et al.  Clin Gastroenterol Hepatol 2022;20: e1378-87 |
| Germany | CP was diagnosed if two or more of the following were present: (i) a typical history of recurrent pancreatitis, (ii) pancreatic calcifications, and (iii) pancreatic ductal irregularities demonstrated by ERCP, MRI, or characteristic sonographic findings. | PEI was diagnosed if the fecal elastase-1 concentration was <200 µg/g stool. |  |
| Poland | CP was diagnosed if any of the following were present in association with abnormalities on imaging studies: (i) abdominal pain consistent with a pancreatic origin, (ii) PEI, or (iii) pancreatic endocrine insufficiency. | PEI was diagnosed if the fecal elastase-1 concentration was <200 µg/g stool, or ^13^C-mixed triglyceride breath test yielded abnormal results. | Morinville VD, et al. J Pediatr Gastroenterol Nutr. 2012;55: 261-5. |
| India | CP was diagnosed if any of the following were present in association with abnormalities on imaging studies: (i) abdominal pain consistent with a pancreatic origin, (ii) PEI, or (iii) pancreatic endocrine insufficiency. | PEI was diagnosed if the fecal elastase-1 concentration was <100 µg/g stool. |  |
| BT-PABA, N-benzoyl-l-tyrosyl-p-aminobenzoic acid; CP, chronic pancreatitis; CT, computer tomography; ERCP, endoscopic retrograde cholangiopancreatography; EUS, endoscopic ultrasonography; MRCP, magnetic resonance cholangiopancreatography; MRI, magnetic resonance imaging; PEI, pancreatic exocrine insufficiency | | | |

| **Supplementary Table 4. Sequences of primers used for genomic PCR** | | |
| --- | --- | --- |
| Target | Forward primer | Reverse primer |
| *Trpv6* | 5’-GGTTGATGTTTGGGGTTAGACTT-3’ | 5’-CAGTCCTGGCCATAGGGTGGAG-3’ |
| PCR conditions: 94 °C for 1 min; 32 cycles of 94 °C for 30 s, 64 °C for 30 s, and 72 °C for 30 s; final extension at 72 °C for 7 min. | | |

| **Supplementary Table 5. Histological scoring for pancreatitis** | | |
| --- | --- | --- |
| Parameter | Score | Indication |
| Edema | 0 | absent |
|  | 1 | focally increased between lobules |
|  | 2 | diffusely increased between lobules |
|  | 3 | acini disrupted and separated |
|  |  |  |
| Inﬂammatory cell inﬁltrate | 0 | absent |
|  | 1 | rare or around ductal margins |
|  | 2 | in the parenchyma (<50% of the lobules) |
|  | 3 | in the parenchyma (>50% of the lobules) |
|  |  |  |
| Necrosis | 0 | absent |
|  | 1 | architectural changes, picnotic nuclei |
|  | 2 | focal necrosis (<10% of the parenchyma) |
|  | 3 | diffuse parenchymal necrosis  (>10% of the parenchyma) |
| Adopted from Moreno C, et al. Am J Physiol Gastrointest Liver Physiol 2006;291: G089-99 | | |

| **Supplementary Table 6. Reagents used to culture pancreatic organoids** | |
| --- | --- |
| Name | Supplier and location |
| B-27 supplement | Gibco, Thermo Fisher Scientific, Waltham, MA |
| Gastrin | Sigma-Aldrich, St. Louis, MO |
| N-acetylcysteine | Sigma-Aldrich, St. Louis, MO |
| Nicotinamide | Sigma-Aldrich, St. Louis, MO |
| Mouse epidermal growth factor | Gibco, Thermo Fisher Scientific, Waltham, MA |
| hNoggin | PeproTech, Rocky Hill, NJ |
| Fibroblast growth factor-10 | PeproTech, Rocky Hill, NJ |
| R-spondin conditioned medium | Prepared in-house |
| Afamin/Wnt3a conditioned medium | MBL, Nagoya, Japan |

| **Supplementary Table 7. Primer sequences used for real-time PCR** | | |
| --- | --- | --- |
| Target | Forward primer | Reverse primer |
| *Slc9a1* | 5’-TCATCCACCTCGGATCTTCCC-3’ | 5’-TCCTGAGAACAGGTAGCAGTC-3’ |
| *Slc4a4* | 5’-GATGCCACCGACAACATGC-3’ | 5’-TCAAGATGGTAAGCGGTTGAC-3’ |
| *Prss1* | 5’-CATCCAAGTGAGATTGGGGGA-3’ | 5’-GGTCTTCCTATTGAAGTTGGGG-3’ |
| *Spink1* | 5’-TTTGGCCCTGCTGAGTTTAGC-3’ | 5’-TGGCATAAGTAATTCCGTCAGTC-3’ |
| *Trpv6* | 5’-ACTGTGATGTTAGAGCGGAAGCTAC-3’ | 5’-GTAGGAAAGGACAGATAGGCACCAAA-3’ |
| *β-actin* | 5’-GGCTGTATTCCCCTCCATCG-3’ | 5’-CCAGTTGGTAACAATGCCATGT-3’ |

| **Supplementary Table 8. Comparison of clinical outcomes between 94 patients with *TRPV6*-related pancreatitis and genotype-stratified patient groups** | | | |
| --- | --- | --- | --- |
| Clinical outcomes | *TRPV6*- vs. *PRSS1*-related | *TRPV6-* vs. *SPINK1*-related | *TRPV6-* vs. PV-negative |
| Age at symptom onset | <0.001 | 0.77 | <0.001 |
| Pancreatic calcification | 0.16 | 0.07 | <0.001 |
| PEI | 0.002 | 0.56 | <0.001 |
| DM | 0.16 | 0.64 | 0.006 |
| Endoscopic treatment | 0.21 | 0.12 | <0.001 |
| Surgery | 0.002 | 0.21 | 0.33 |
| All intervention | 0.45 | 0.18 | <0.001 |
| *P* values for the comparisons are presented. DM, diabetes mellitus; PEI, pancreatic exocrine insufficiency; PV, pathogenic variant | | | |

| **Supplementary Table 9. Clinical outcomes of 80 patients with *TRPV6*-related pancreatitis, excluding double heterozygotes for *TRPV6* and *SPINK1* variants** | |
| --- | --- |
|  | ***TRPV6*-related**  **(n = 80)** |
| Sex, male, n (%) | 48 (60.0) |
| CP/RAP | 57/23 |
| Etiology, n (%) |  |
| Idiopathic | 52 |
| Hereditary/Familial | 28 |
| Pancreas divisum | 0 |
| Median age at last follow-up (95% CI) | 26 (21.1–30.9) |
| Onset of symptoms |  |
| Yes, n (%) | 75 (93.8) |
| Median age at symptom onset, years (95% CI) | 15 (12.1–17.9) |
| Pancreatic calcification |  |
| Yes, n (%) | 39 (48.8) |
| Median age at diagnosis, years (95% CI) | 29 (25.1–32.9) |
| Pancreatic exocrine insufficiency |  |
| Yes, n (%) | 19 (23.8) |
| Median age at diagnosis, years (95% CI) | Not reached |
| Diabetes mellitus |  |
| Yes, n (%) | 14 (17.5) |
| Median age at diagnosis, years (95% CI) | 52.0 (39.4–64.6) |
| Intervention for pancreatitis |  |
| Yes, n (%) | 28 (35.0) |
| Median age at diagnosis, years (95% CI) | 37 (26.0–48.0) |
| Endoscopic treatment |  |
| Yes, n (%) | 25 (31.3) |
| Median age at the first treatment, years (95% CI) | Not reached |
| Surgery |  |
| Yes, n (%) | 8 (10.0) |
| Median age at pain onset, years (95% CI) | Not reached |
| Pancreatic cancer |  |
| Yes, n (%) | 0 (0) |
| Median age at diagnosis, years (95% CI) | Not reached |
| CI, confidence intervals; CP, chronic pancreatitis; RAP, recurrent acute pancreatitis. | |

| **Supplementary Table 10. Comparison of clinical outcomes across pathogenic genotypes, excluding 14 double heterozygous cases for *TRPV6* and *SPINK1* variants** | | | |
| --- | --- | --- | --- |
| Clinical outcomes | *TRPV6-* vs. *PRSS1*-related | *TRPV6-* vs. *SPINK1*-related | *TRPV6-* vs. PV-negative |
| Age at onset | 0.003 | 0.85 | <0.001 |
| Pancreatic calcification | 0.09 | 0.03 | <0.001 |
| Pancreatic exocrine insufficiency | 0.005 | 0.66 | <0.001 |
| Diabetes mellitus | 0.23 | 0.76 | 0.005 |
| Endoscopic treatment | 0.48 | 0.041 | 0.006 |
| Surgery | 0.004 | 0.24 | 0.36 |
| All intervention | 0.22 | 0.063 | 0.001 |
| *P* values for the comparisons are presented. PV, pathogenic variant | | | |
