# Supplementary material for: TRPV6-related pancreatitis: natural history and the impact of the pancreas-specific deletion on pancreatitis in mice
Source: J Gastroenterol. 2025 Nov 17;61(2):207–21. doi: 10.1007/s00535-025-02323-y (PMC12924865; doi:10.1007/s00535-025-02323-y)
Supplement: Supplementary file 2 — Supplementary file2 (DOCX 36200 KB) [file 535_2025_2323_MOESM2_ESM.docx]

**Supplementary Figure 1. Functional analysis of novel non-synonymous *TRPV6* variants by Ca²⁺ imaging assay.** TRPV6 activity was assessed in HEK293 cells expressing the variants using a Ca²⁺ imaging assay. Data represent mean + standard error (number of assays, 17–54) from the sum of 2 or 3 independent transfections. The Δ*F_340_*/*F_380_* value in cells expressing wild-type TRPV6 was set as 100% TRPV6 activity. **: *P* < 0.01 vs. wild-type.

**Supplementary Figure 2. Minigene assay to assess the splicing outcome of the c.347-2A>G variant.** (**A**) Agarose gel electrophoresis of reverse transcription-PCR products from HEK293T cells transfected with wild-type or variant minigene constructs. The c.347-2A>G construct yielded a slightly larger fragment than the wild-type. sm, size marker (100-bp ladder). (**B**) Sequencing of gel-purified reverse transcription-PCR products demonstrated retention of the last 33 bp of intron 2 between exon 2 and exon 3. (**C**) Schematic representation of the splicing alteration caused by c.347-2A>G. Disruption of the consensus acceptor site activated a cryptic acceptor site within intron 2, resulting in the 33-bp retention. The mutated nucleotide is shown in red. (**D**) The predicted mutant protein is truncated, with deletion of 649 amino acids and insertion of two additional residues.

**Supplementary Figure 3. Minigene assay evaluating the splicing effect of the c.469+1G>C variant.** (**A**) Agarose gel electrophoresis of RT-PCR products from HEK293T cells transfected with wild-type or variant minigene constructs. The c.469+1G>C construct yielded a shorter fragment than the wild-type. sm, size marker (100-bp ladder). (**B**) Sequencing of gel-purified reverse transcription-PCR products confirmed skipping of the entire exon 3 in the c.469+1G>C variant. (**C**) Schematic representation of exon 3 (123 bp) skipping caused by c.469+1G>C. The mutated nucleotide is shown in red. (**D**) The predicted mutant protein harbors an in-frame deletion of 41 amino acids, including the ANK2 region.

**Supplementary Figure 4. Minigene assay evaluating the splicing effect of the c.607+5G>C variant.** (**A**) Agarose gel electrophoresis of reverse transcription-PCR products from HEK293T cells transfected with wild-type or variant minigene constructs. The c.607+5G>C construct yielded a smaller fragment than the wild-type. sm, size marker (100-bp ladder). (**B**) Sequencing of gel-purified reverse transcription-PCR products demonstrated partial, but not complete, inclusion of exon 4 in the c.607+5G>C variant. (**C**) Schematic representation of the splicing alteration. Disruption of the consensus donor site activated a cryptic donor site within exon 4, resulting in partial exon 4 deletion. The mutated nucleotide is shown in red. (**D**) The predicted mutant protein is truncated, carrying a Val173Gly substitution followed by nine novel amino acids and premature termination.

**Supplementary Figure 5. Minigene assay evaluating the splicing effect of the c.1029+1G>A variant.** (**A**) Agarose gel electrophoresis of reverse transcription-PCR products from HEK293T cells transfected with wild-type or variant minigene constructs. The c.1029+1G>A construct yielded a longer fragment than the wild-type. sm, size marker (100-bp ladder). (**B**) Sequencing of gel-purified reverse transcription-PCR products revealed complete retention of intron 7 between exon 7 and exon 8 in the c.1029+1G>A variant. (**C**) Schematic representation of intron 7 retention caused by c.1029+1G>A. The mutated nucleotide is shown in red. (**D**) The predicted mutant protein is truncated, carrying an Ala344Ile substitution followed by 34 novel amino acids.

**Supplementary Figure 6. Minigene assay evaluating the splicing effect of the c.1407-2A>G variant.** (**A**) Agarose gel electrophoresis of reverse transcription-PCR products from HEK293T cells transfected with wild-type or variant minigene constructs. The c.1407-2A>G construct yielded a shorter fragment than the wild-type. sm, size marker (100-bp ladder). (**B**) Sequencing of gel-purified reverse transcription-PCR products confirmed skipping of the entire exon 11 in the c.1407-2A>G variant. (**C**) Schematic representation of exon 11 skipping caused by c.1407-2A>G. The mutated nucleotide is shown in red. (**D**) The predicted mutant protein is truncated due to a premature stop codon at position 470.

**Supplementary Figure 7. Minigene assay evaluating the splicing effect of the c.2015+2T>C variant.** (**A**) Agarose gel electrophoresis of reverse transcription-PCR products from HEK293T cells transfected with wild-type or variant minigene constructs. The c.2015+2T>C construct yielded a slightly longer fragment than the wild-type. sm, size marker (100-bp ladder). (**B**) Sequencing of gel-purified reverse transcription-PCR products revealed retention of the first 17 bp of intron 14 between exon 14 and exon 15 in the c.2015+2T>C variant. (**C**) Schematic representation of the splicing alteration. Disruption of the consensus donor site activated a cryptic donor site in intron 14, resulting in 17-bp retention. The mutated nucleotide is shown in red. (**D**) The predicted mutant protein is truncated, containing a Val673Arg substitution followed by 39 novel amino acids.


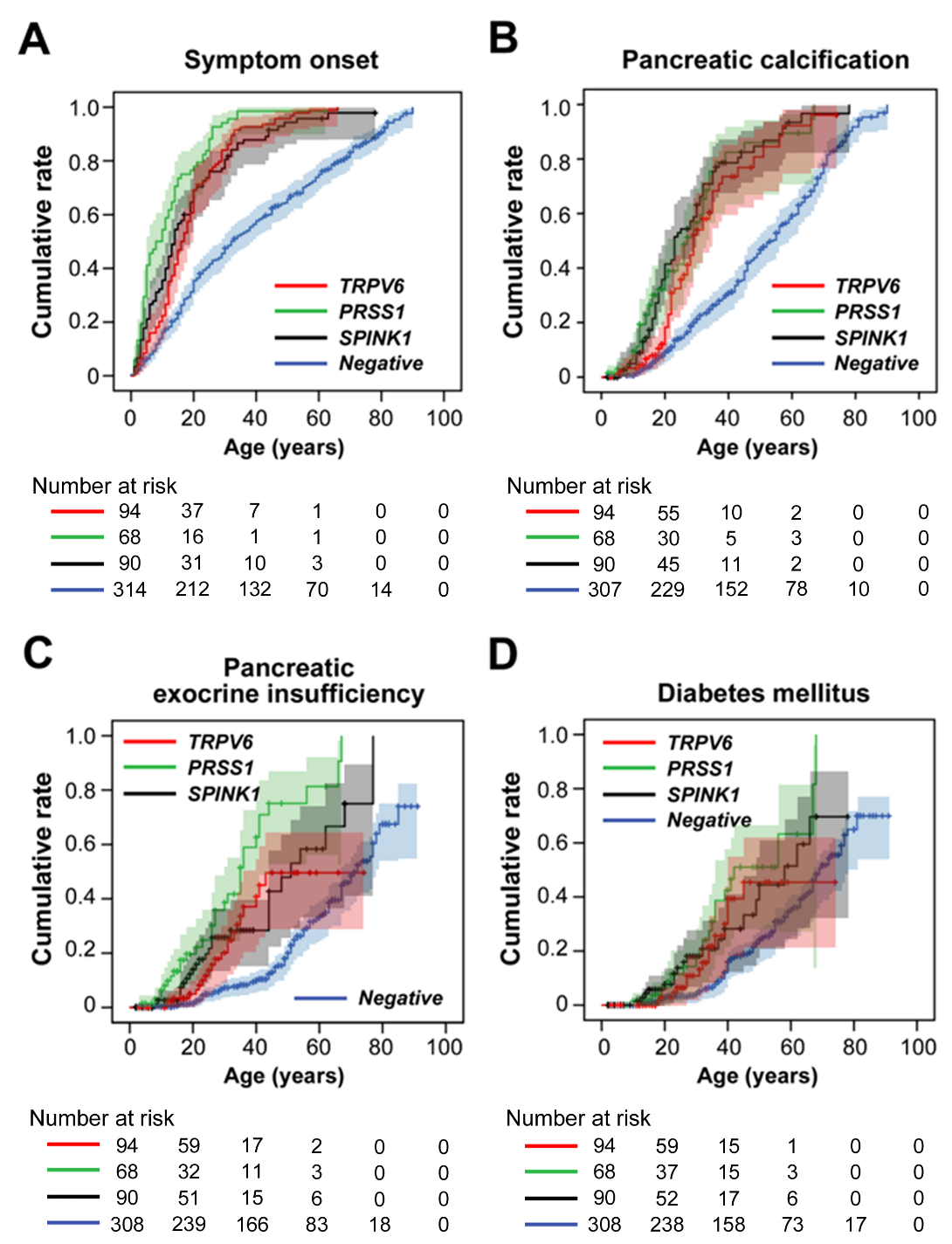


**Supplementary Figure 8. Comparison of clinical outcomes across pathogenic genotypes.** Kaplan–Meier plots show cumulative rates of (**A**) symptom onset, (**B**) pancreatic calcification, (**C**) pancreatic exocrine insufficiency, and (**D**) diabetes mellitus, stratified by genotype (*TRPV6*-related, *PRSS1*-related, *SPINK1*-related, or PV-negative pancreatitis). Shaded areas indicate 95% confidence intervals. Censored cases are indicated by tick marks on the curves. PV: pathogenic variant.


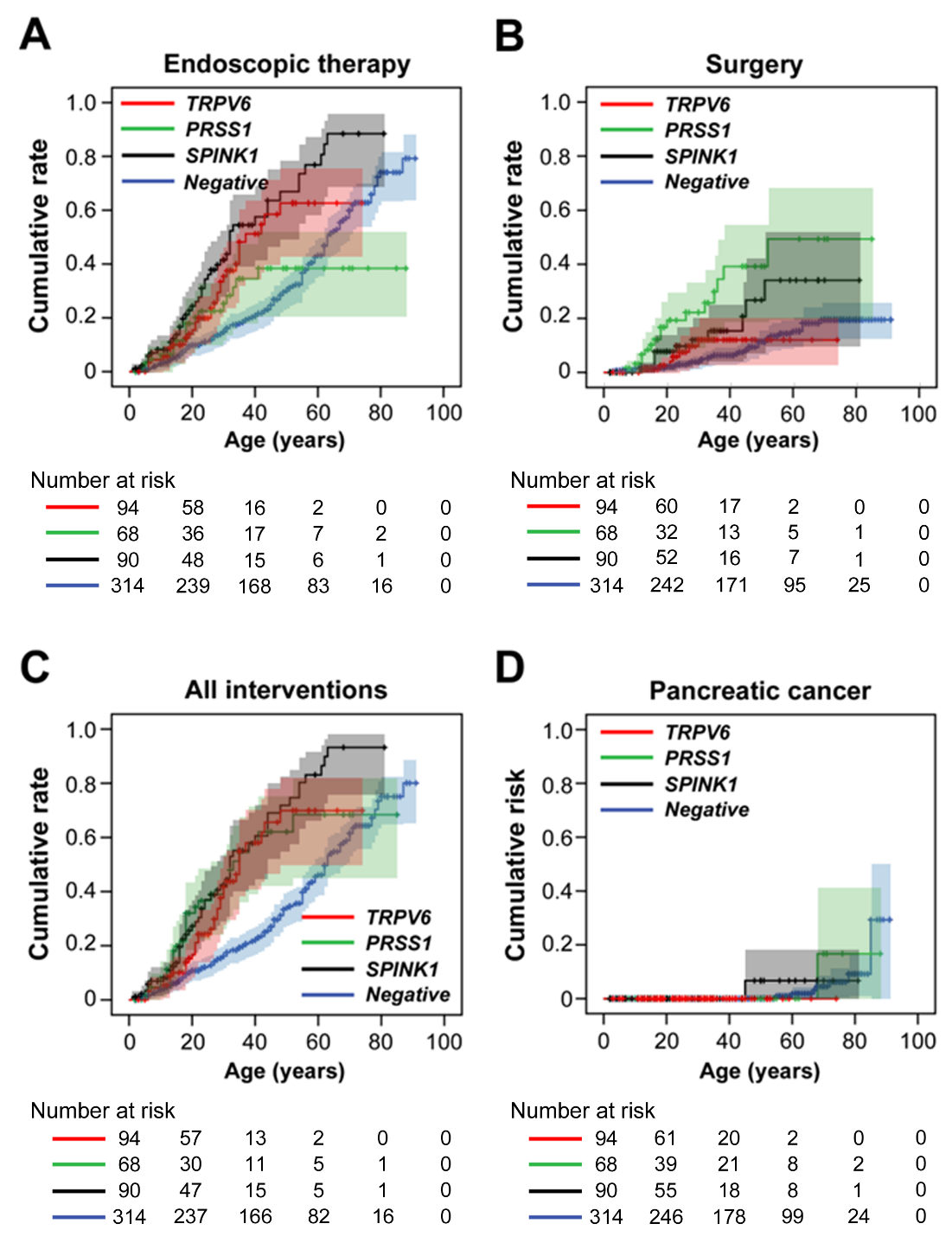


**Supplementary Figure 9. Timing of first interventions for pancreatitis and pancreatic cancer diagnosis across pathogenic genotypes.** Kaplan–Meier plots show cumulative rates of (**A**) endoscopic treatment, (**B**) surgery, (**C**) all interventions, and (**D**) the diagnosis of pancreatic cancer stratified by genotype (*TRPV6*-related, *PRSS1*-related, *SPINK1*-related, or PV-negative pancreatitis). Shaded areas indicate 95% confidence intervals. Censored cases are indicated by tick marks on curves. PV: pathogenic variant.

**Supplementary Figure 10. Comparison of clinical outcomes across pathogenic genotypes, excluding double-heterozygous cases.** Kaplan–Meier plots show cumulative rates of (**A**) symptom onset, (**B**) pancreatic calcification, (**C**) pancreatic exocrine insufficiency, and (**D**) diabetes mellitus, stratified by genotype (*TRPV6*-related, *PRSS1*-related, *SPINK1*-related, or PV-negative pancreatitis). Fourteen double-heterozygous cases (*TRPV6* and *SPINK1*) were excluded from the analysis. Censored cases are indicated by tick marks on curves. PV: pathogenic variant.

**Supplementary Figure 11. Timing of first interventions for pancreatitis and pancreatic cancer diagnosis across pathogenic genotypes, excluding double-heterozygous cases.** Kaplan–Meier plots show cumulative rates of (**A**) endoscopic treatment, (**B**) surgery, (**C**) all interventions, and (**D**) the diagnosis of pancreatic cancer stratified by genotype (*TRPV6*-related, *PRSS1*-related, *SPINK1*-related, or PV-negative pancreatitis). Fourteen double-heterozygous cases (*TRPV6* and *SPINK1*) were excluded from the analysis. Censored cases are indicated by tick marks on curves. PV: pathogenic variant.

**Supplementary Figure 12. Establishment of a pancreas-specific *Trpv6* knockout mouse.**(**A**) Schema of the *Trpv6* floxed allele and *Cre*-mediated pancreas-specific recombination. Black rectangles indicate exons; black triangles indicate *loxP* sequence. Recombination by *Cre* removes exons 13–15 in the *Trpv6* conditional knockout (CKO) mouse. (**B**) Genomic DNA was extracted from the lung, heart, liver, and pancreas of *Trpv6* CKO mouse. PCR amplified the region spanning exons 13–15. In the pancreas, a shorter PCR product (528 bp) indicated successful recombination, whereas the floxed allele (3,771 bp) was detected in the lung, heart, and liver. sm, size marker (100-bp ladder). (**C**) Representative H&E staining of the pancreas. No significant histological changes were observed in Trpv6 CKO mice up to 90 days of age, similar to floxed controls. Black bar = 100 μm.

**Supplementary Figure 13. Comparison of histological findings of the pancreas.** *Trpv6* floxed mice and pancreas-specific *Trpv6* CKO mice received eight hourly intraperitoneal caerulein injections (100 μg/kg body weight) or saline for two consecutive days. Histological findings of the pancreas, harvested at 32 hours after the first caerulein or saline injection, were quantified in *Trpv6* floxed (*n* = 35 high-power fields from 7 mice) and *Trpv6* CKO mice (*n* = 20 high-power fields from 4 mice). **: *P* < 0.001 vs. *Trpv6* floxed mice.

**Supplementary Figure 14. *Trpv6* deletion had no significant impact on pancreatic organoids. (A)** Pancreatic organoids were established from *Trpv6* floxed and CKO mice. (**B**) Expression levels of ductal markers (*Slc9a1*, *Slc4a4*) and acinar markers (*Prss1*, *Spink1*) were assessed by real-time PCR (*n* = 4). (**C, D**) Organoids were treated with 10 μmol/L forskolin for 16 hours, and their diameters were measured in six randomly selected areas. The enlargement ratio was calculated as: (cross-sectional area after forskolin treatment) / (cross-sectional area before treatment). White bar = 500 μm. (**E, F**) Organoids were cultured in medium containing the indicated Ca²⁺ concentrations for 36 hours. Enlargement ratios were calculated and compared between *Trpv6* floxed and CKO organoids (*n* = 4 for each Ca²⁺ concentration). n.s., not significant; n.d., not detectable.

**Supplementary Figure 15. TRPV6 expression was upregulated by caerulein treatment.**

**(A)** *Trpv6* floxed mice and pancreas-specific *Trpv6* CKO mice received eight intraperitoneal injections of caerulein (100 μg/kg body weight) for two consecutive days. Mice were euthanized at 32 hours after the first injection, and pancreatic TRPV6 expression was evaluated by immunohistochemistry using an anti-TRPV6 antibody. Corresponding H&E images are also shown. (**B**) Eight-week-old male C57BL/6J mice received eight hourly intraperitoneal injections of caerulein (100 μg/kg body weight) for one or two consecutive days. Mice were euthanized at 8 or 32 hours, and pancreatic tissues were harvested. Total RNA was extracted and reverse-transcribed, and the mRNA levels of *Trpv6* and *β-actin* were quantified by real-time PCR. *n* = 4 per time point. **: *P* < 0.01 vs. control (saline).
